# Reciprocal control of motility and biofilm formation by the PdhS2 two-component sensor kinase of *Agrobacterium tumefaciens*

**DOI:** 10.1099/mic.0.000758

**Published:** 2019-01-08

**Authors:** Jason E. Heindl, Daniel Crosby, Sukhdev Brar, John F. Pinto, Tiyan Singletary, Daniel Merenich, Justin L. Eagan, Aaron M. Buechlein, Eric L. Bruger, Christopher M. Waters, Clay Fuqua

**Affiliations:** ^1^​ Department of Biology, Indiana University, Bloomington, IN 47405, USA; ^2^​ Department of Biological Sciences, University of the Sciences in Philadelphia, Philadelphia, PA 19104, USA; ^3^​ Center for Genomics and Bioinformatics, Indiana University, Bloomington, IN 47405, USA; ^4^​ Department of Microbiology and Molecular Genetics, Michigan State University, East Lansing, MI 48824, USA; ^†^​ Present address: Department of Biological Sciences, Carnegie Mellon University, Pittsburgh, PA 15213, USA.; ^‡^​ Present address: Department of Biological Sciences, University of Idaho, Moscow, ID 83844, USA.

**Keywords:** development, sensor kinase, phosphorelay, *Agrobacterium tumefaciens*, biofilm, motility

## Abstract

A core regulatory pathway that directs developmental transitions and cellular asymmetries in *Agrobacterium tumefaciens* involves two overlapping, integrated phosphorelays. One of these phosphorelays putatively includes four histidine sensor kinase homologues, DivJ, PleC, PdhS1 and PdhS2, and two response regulators, DivK and PleD. In several different alphaproteobacteria, this pathway influences a conserved downstream phosphorelay that ultimately controls the phosphorylation state of the CtrA master response regulator. The PdhS2 sensor kinase reciprocally regulates biofilm formation and swimming motility. In the current study, the mechanisms by which the *A. tumefaciens* sensor kinase PdhS2 directs this regulation are delineated. PdhS2 lacking a key residue implicated in phosphatase activity is markedly deficient in proper control of attachment and motility phenotypes, whereas a kinase-deficient PdhS2 mutant is only modestly affected. A genetic interaction between DivK and PdhS2 is revealed, unmasking one of several connections between PdhS2-dependent phenotypes and transcriptional control by CtrA. Epistasis experiments suggest that PdhS2 may function independently of the CckA sensor kinase, the cognate sensor kinase for CtrA, which is inhibited by DivK. Global expression analysis of the *pdhS2* mutant reveals a restricted regulon, most likely functioning through CtrA to separately control motility and regulate the levels of the intracellular signal cyclic diguanylate monophosphate (cdGMP), thereby affecting the production of adhesive polysaccharides and attachment. We hypothesize that in *A. tumefaciens* the CtrA regulatory circuit has expanded to include additional inputs through the addition of PdhS-type sensor kinases, likely fine-tuning the response of this organism to the soil microenvironment.

## Introduction

Bacteria are sometimes considered to be elementary life forms, with simple body plans, streamlined reproductive cycles and monolithic behaviour when compared with higher eukaryotes. However, many bacteria can exhibit a remarkable diversity of developmental complexity, both temporal and morphological [1, 2]. Even bacterial species whose cells appear to be morphologically uniform, such as rod-shaped *Escherichia coli* or coccoid *Staphylococcus aureus ,* possess distinct cellular architectures, as well as intricately timed cell division programs, and a large number of bacteria can form multicellular biofilms [3, 4]. Developmental processes in bacteria, as in higher eukaryotes, are driven by factors that may be considered both cell-intrinsic and cell-extrinsic. Intrinsic factors include genomic and proteomic content, while extrinsic factors comprise environmental conditions, such as pH and temperature, which cells sense and to which they respond [5].

Members of the *Alphaproteobacteria* group include host-associated pathogens (e.g. *Brucella* sp., *Bartonella* sp.), host-associated commensals (e.g. *Sinorhizobium* sp., *Bradyrhizobium* sp.), and free-living aquatic and marine bacteria (e.g. *Caulobacter* sp., *Rhodobacter* sp., *Ruegeria* sp.). It is now recognized that several alphaproteobacteria divide asymmetrically, during which cells elongate, duplicate and segregate their genomic content between two non-equivalent compartments of predivisional cells, and finally generate two cells by cytokinesis [6, 7]. Notably, cellular components are unevenly distributed between the two daughter cells during cell division, including surface structures (e.g. flagella and polar polysaccharides), cell wall components (e.g. peptidoglycan) and even cytoplasmic complexes (e.g. heat shock proteins). For example, there may be a clear segregation of existing organelles to one daughter cell, while the second cell generates these structures *de novo* [6, 8–10]. Although the specific details may vary among different taxa, the end result is the production of a young daughter cell and a comparatively oldmother cell. Not only does this uneven division partition senescence among the products of cell division, but it also allows for the generation of functionally distinct cell types. For example, in *Caulobacter crescentus* the non-motile stalked cell type can attach to surfaces using its polar adhesin, which is called the holdfast [11]. This stalked cell then serves as the mother cell during multiple rounds of cell division, generating and releasing motile swarmer cells upon each cytokinetic event [12]. Motile swarmer cells are prohibited from entering the cell division cycle until differentiation into the non-motile stalked form [13, 14].

**Fig. 1.**
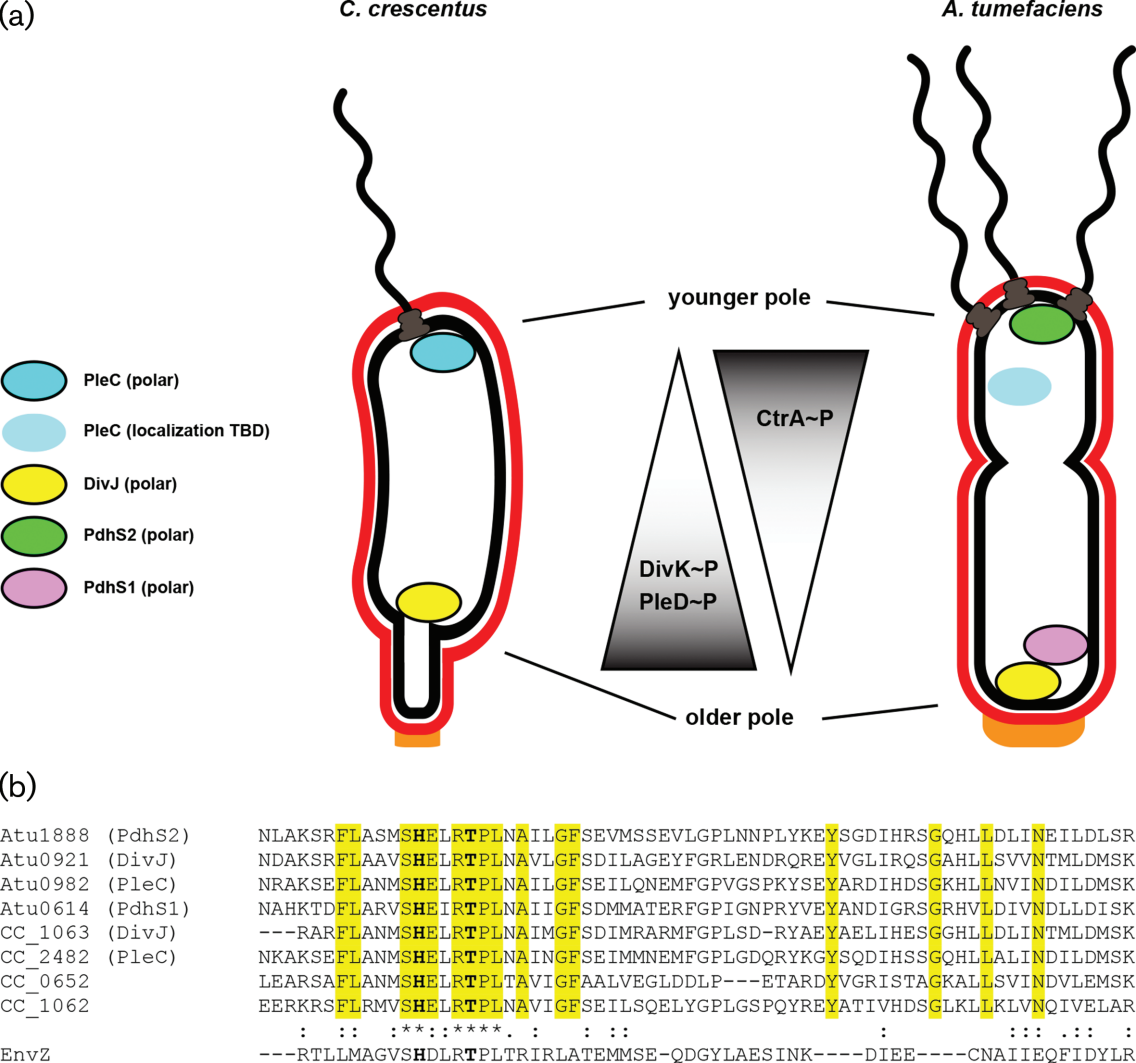
The PdhS kinases of *C. crescentus* and *A. tumefaciens* localize differentially and affect phenotypic outputs through the response regulators DivK, PleD and CtrA. (a) Cartoon model of known localization of the namesake PdhS kinases from *C. crescentus *, PleC and DivJ, and three PdhS kinases from *A. tumefaciens *, PdhS1, PdhS2 and DivJ. Kinases represented as coloured ovals with a black border experimentally localize to the indicated poles. The PleC oval without a border has not been experimentally demonstrated to localize in *A. tumefaciens *. As a result of this localization, the phosphorylation status of the direct PdhS kinase targets, DivK and PleD, and the indirect target, CtrA, may be differentially affected. (b) Multiple sequence alignment of the HisKA domain from the PdhS kinases of *C. crescentus* and *A. tumefaciens *. Sequences were aligned using the Clustal Omega web service hosted by the European Molecular Biology Laboratory (EMBL) – European Bioinformatics Institute. The four PdhS kinases from *A. tumefaciens* plus PleC and DivJ from *C. crescentus* were included. Also included were two additional predicted PdhS kinases, CC_0652 and CC_1062, from *C. crescentus *. The EnvZ sensor kinase is included for comparison. Yellow highlighting indicates residues that define the PdhS kinases. The conserved histidine and threonine residues mutated in this work are in bold.

Underlying asymmetric cell division is subcellular differentiation that includes the localization of specific regulatory proteins to programmed locations within each cell [15]. Prominent among these in many alphaproteobacteria are the components of two overlapping phosphorelays, the first of which functions through the response regulators DivK and PleD (the DivK–PleD relay) and the second of which functions primarily through the response regulator CtrA (the CtrA relay). The pathways are connected through DivK, which controls the initiation of the CtrA relay by regulating its cognate sensor kinase CckA [16, 17]. Collectively we refer to these two relays as the DivK–CtrA pathway. In the well-studied *C. crescentus* system the membrane-associated sensor histidine kinases PleC and DivJ control the phosphorylation state of DivK and PleD, and localize to opposing poles of the predivisional cell [18–21]. Through antagonistic kinase and phosphatase activities on their target response regulators, DivK and PleD, PleC and DivJ inversely manifest their activity on the most downstream component of the DivK–CtrA pathway, the response regulator CtrA [22–25]. DivJ is retained at the stalked cell pole and serves as a DivK/PleD kinase, increasing the DivK ~P concentration and thereby indirectly diminishing CtrA ~P levels in this region of the cell (Fig. 1a). Conversely, PleC localizes to the pole distal to the stalk, where the single polar flagellum is assembled, dephosphorylating DivK, and ultimately leading to increased CtrA ~P levels and activity. Phospho-CtrA binds to the replication origin, thereby preventing DNA replication, and also acts as a transcriptional regulator for many genes, including activating those for assembly of the flagellum and motility [26–28]. The CtrA relay is also influenced by the DivK–PleD relay through the levels of the second messenger cyclic diguanylate monophosphate (cdGMP). DivJ-dependent phosphorylation of PleD at the stalk pole of the predivisional cell stimulates its diguanylate cyclase activity, resulting in higher levels of cdGMP at this end of the cell. The CckA kinase that initiates the CtrA relay is also biased away from its kinase and towards its phosphatase activity by direct allosteric control through high levels of cdGMP, thereby reinforcing a CtrA ~P gradient, which is relatively low at the stalk pole and increasing towards the distal pole [29–34] (Fig. 1a).

*Agrobacterium tumefaciens* is a plant pathogen of the class *Alphaproteobacteria* that is not stalked, but like *C. crescentus *, divides asymmetrically, generating a motile daughter cell from a mother cell [6]. As a facultative pathogen, *A. tumefaciens *’s lifestyle differs substantially from that of the freshwater oligotroph *C. crescentus *. Nonetheless, core components of the DivK–CtrA pathway are well conserved in *A. tumefaciens *, including three non-essential PleC/DivJ homologue sensor kinase (PdhS) homologues, PleC (Atu0982), PdhS1 (Atu0614) and PdhS2 (Atu1888). The *divJ* gene (Atu0921) is essential in *A. tumefaciens* [35, 36]. We have previously shown that the three non-essential PdhS homologues have distinct roles in the normal cellular development of *A. tumefaciens* [36]. Mutants in PleC and PdhS1, as well as the *A. tumefaciens* DivK homologue, all manifested marked effects on cell division, with branched and elongated cells, as well as deficiencies in motility and biofilm formation. To date, the essentiality of *divJ* has precluded exhaustive phenotypic analysis of its role [36]. The fourth PdhS family member, PdhS2, does not appear to participate in the regulation of cell division, as all cells are morphologically wild-type in appearance [36]. Loss of PdhS2, however, results in dramatically increased attachment and biofilm formation, and a simultaneous dramatic reduction in motility. Reciprocal regulation of these phenotypes is often a hallmark of regulation by cdGMP [37]. In this work we further explore the mechanism by which PdhS2 regulates attachment and motility. Our results genetically connect DivK with PdhS2 and transcriptional profiling clearly implicates CtrA as their downstream regulatory effector. We also show a clear intersection of PdhS2 activity and the activity of several diguanylate cyclases, suggesting that PdhS2 and cdGMP coordinately regulate biofilm formation and motility in *A. tumefaciens *. Collectively, our findings suggest that PdhS2 activity is specifically required for the proper development of motile daughter cells.

## Methods

### Strains and plasmids

The bacterial strains, plasmids and oligonucleotides used in these studies are listed in Tables S1–S3. *A. tumefaciens* was routinely cultivated at 28 °C in AT minimal medium plus 1 % (w/v) glucose as a carbon source and 15 mM (NH_4_)_2_SO_4_ as a nitrogen source (ATGN), without exogenous FeSO_4_. For biofilm assays, 22 µM FeSO_4_ was included in the media. *E. coli* was routinely cultivated at 37 °C in lysogeny broth (LB). Antibiotics were used at the following concentrations (* A. tumefaciens */* E. coli *): ampicillin (100/100 µg ml^−1^), kanamycin (Km; 150/25 µg ml^−1^), gentamicin (150/30 µg ml^−1^), spectinomycin (300/100 µg ml^−1^) and tetracycline (4/10 µg ml^−1^).

Non-polar, markerless deletion of *pdhS2* (Atu1888) in all of the genetic backgrounds used in this work was accomplished using splicing by overlap extension (SOE) polymerase chain reaction (PCR) followed by homologous recombination, as described in [36]. Suicide plasmid pJEH040 carries an approximately 1 kb SOE deletion fragment of *pdhS2* on a pNPTS138 vector backbone. pNPTS138 is a ColE1 plasmid and as such is unable to replicate in *A. tumefaciens *. pJEH040 was delivered to recipient strains by either transformation or conjugation followed by selection on ATGN plates supplemented with 300 µg ml^−1^ Km, selecting for *A. tumefaciens* cells in which pJEH040 had integrated at the chromosomal *pdhS2* locus by homologous recombination. Recombinants were then grown overnight at 28 °C in ATGN in the absence of Km and plated the following day onto ATGN with sucrose substituted for glucose (ATSN) agar plates to select for sucrose resistant (Suc^R^) allelic replacement candidates. After 3 days’ growth at 28 °C, the colonies were patched in parallel onto ATGN Km and ATSN plates. Km^S^ Suc^R^ recombinants were then tested for the targeted deletion by diagnostic PCR using primers external to the *pdhS2* locus (JEH100 and JEH113) as well as internal primers (JEH85 and JEH87). Candidate colonies were further streak-purified and verified a second time by diagnostic PCR before being used in downstream assays. Non-polar, markerless deletion of *dgcB* (Atu1691) in the Δ*pleD* and Δ*pdhS2*Δ*pleD* genetic backgrounds was achieved using the above strategy with the pNPTS138 derivative pJX802.

Site-directed mutagenesis of *pdhS2* was achieved using mutagenic primer pairs JEH245/JEH246 (for generating the His271Ala allele, H271A) or JEH261/JEH262 (for generating the Thr275Ala allele, T275A). Plasmid pJEH021 carrying the wild-type *pdhS2* sequence was amplified by PCR using the above primer pairs. Following amplification, reaction mixtures were treated with *Dpn*I restriction endonuclease to remove template plasmid and then transformed into TOP10 F′ *E. coli* competent cells. Purified plasmids from each transformation were sequenced and those containing the desired mutations, pJEH091 for His271Ala and pJEH099 for Thr275Ala, were selected for sub-cloning. pJEH091 and pJEH099 were digested with *Nde*I and *Nhe*I followed by gel electrophoresis and purification of the resulting insert. The inserts were ligated into similarly digested pSRKGm and transformed into competent *E. coli* TOP10 F′ cells. Purified plasmids from each transformation were sequenced to verify their identity. The resulting plasmids, pJEH092 (H271A) and pJEH102 (T275A), were used to transform *A. tumefaciens *. To generate a PdhS2 allele carrying both H271A and T275A mutations, the same steps were followed as above using plasmid pJEH091 as a template with mutagenic primers JEH261/JEH262.

Translational fusions of full-length wild-type PdhS2 and DivJ to GFP were constructed as follows. *pdhS2* and *divJ*, each lacking a stop codon, were amplified by PCR using primer pairs JEH65/JEH146 (*pdhS2*) and JEH147/JEH148 (*divJ*) with *A. tumefaciens* strain C58 genomic DNA as a template. The primer design for these amplifications included 5′ *Nde*I and 3′ *Nhe*I restriction sites. The *gfpmut3* gene including a 5′ *Nhe*I site and a 3′ *Kpn*I site was amplified using primer pair JEH149/JEH150 and pJZ383 as a template. Amplicons were gel-purified, ligated into pGEM-T Easy, transformed into competent TOP10 F′ *E. coli *, and eventually sequenced. The resulting plasmids, pJEH052 (*pdhS2*), pJEH053 (*gfpmut3*) and pJEH054 (*divJ*), were digested with either *Nde*I and *Nhe*I (pJEH052 and pJEH054) or *Nhe*I and *Kpn*I (pJEH053). The inserts were gel-purified and used in a three-component ligation with *Nde*I/*Kpn*I-digested pSRKGm, generating pJEH060 (PdhS2-GFP) and pJEH078 (DivJ-GFP). The sequenced plasmids were used to transform *A. tumefaciens *.

The reporter gene fusion constructs included predicted promoter regions from between 200 bp and 400 bp upstream of the indicated gene through the start codon. Each upstream region was amplified by PCR using the primers listed in Table S3 using *A. tumefaciens* genomic DNA as a template. Amplicons were gel-purified, ligated into pGEM-T Easy, transformed into competent TOP10 F′ *E. coli *, and eventually sequenced. The resulting plasmids, pJEH113 (*ccrM*, Atu0794, promoter), pJEH115 (*ctrA*, Atu2434, promoter) and pJEH119 (*pdhS1*, Atu0614, promoter), were digested with either *Kpn*I and *Hin*DIII (pJEH113 and pJEH119) or *Kpn*I and *Pst*I (pJEH115). The inserts were gel-purified and ligated with similarly cleaved pRA301 containing a promoterless *E. coli lacZ* gene without its own ribosome-binding site. The resulting constructs (pJEH121, pJEH122 and pJEH124) carry *lacZ* translationally fused to the start codon for each gene with transcription and translation driven by the fused upstream region. pJEH121, pJEH122 and pJEH124 were used to transform *A. tumefaciens* for subsequent beta-galactosidase assays. Mutant promoters were synthesized by Genewiz and sub-cloned into pRA301 to generate plasmids pJFP006 (for the Atu3318 locus) and pJEH141 (for *dgcB*).

### Static biofilm assays

Overnight cultures in ATGN were sub-cultured in fresh ATGN to an optical density at 600 nm (OD_600_) of 0.1 and grown with aeration at 28 °C until they reached an OD_600_ of 0.25–0.6. Cultures were diluted to OD_600_ of 0.05 and 3 ml was inoculated into each of 4 wells in a 12-well plate. A single coverslip was placed vertically into each well to submerge approximately half of each coverslip. Plates were incubated in a humidified chamber at 28° for 48 h. Coverslips were removed from each well and rinsed with water, and adherent biomass was stained by immersion for 5 min in a 0.1 % (w/v) crystal violet solution. Adsorbed crystal violet was solubilized by immersion in 1 ml 33 % acetic acid and the absorbance of this solution determined at 600 nm (A_600_) on a Synergy HT multi-detection microplate reader (Bio-Tek). The culture density for each sample was also determined by measuring the OD_600_ of each culture. The data are typically presented as A_600_/OD_600_ ratios normalized to values obtained for the wild-type strain within each experiment. ATGN was supplemented with antibiotics and 250 µM IPTG as appropriate. The final inoculations also included supplemental FeSO_4_ (22 µM). Each mutant was evaluated in three independent experiments, each of which contained three technical replicates.

### Motility assays

Wet mounts of exponentially growing cultures were observed under brightfield optics using a Zeiss Axioskop 40 equipped with an AxioCam MRm monochrome digital camera. Swim plates containing 0.3 % agarose in ATGN, supplemented with 1 mM IPTG and antibiotics when appropriate, were inoculated with a single colony of the indicated strain at a central point and incubated for 7 days at 28 °C. Swim ring diameters were measured daily for 7 days. Each experimental condition was tested in three independent experiments containing three technical replicates.

### Microscopy

Cell morphology and localization of PdhS2-GFP and DivJ-GFP were evaluated using a Nikon E800 fluorescence microscope equipped with a Photometrics Cascade cooled CCD camera. Overnight cultures were grown in ATGN with gentamicin and 250 µM IPTG. The following day each strain was sub-cultured to an OD_600_ of 0.1 and then grown at 28 °C with aeration until it reached anOD_600_ of 0.5–0.8. The culture (0.5 µl) was transferred to a 1 % ATGN/agarose pad on a clean glass slide and a clean 22×22 mm number 1.5 glass coverslip was placed on top. Images were acquired using a 100× oil immersion objective and phase contrast optics or epifluorescence with a FITC-HYQ filter set (Nikon; excitation filter=480/40 nm, dichromatic mirror=505 nm, absorption filter=535/50 nm). Time-lapse microscopy utilized a Nikon Ti-E inverted fluorescence microscope with a Plan Apo 60×/1.40 oil Ph3 DM objective, a DAPI/FITC/Cy3/Cy5 filter cube, an Andor iXon3 885 EMCCD camera and a Lumencor Spectra X solid-state light engine at 20 % power. For time-lapse imaging, the agarose pads included 250 µM IPTG and coverslips were attached to the glass slide using a gas-permeable 1 : 1 : 1 mixture of Vaseline, lanolin and paraffin. Phase and fluorescence images were captured every 20 min for 8 h using a 60 ms (phase) or 2 s (fluorescence) exposure. Images were analysed using ImageJ [38–40].

### Transcriptional profiling

Whole-genome transcriptional profiling using custom 60-mer oligonucleotide microarrays was performed essentially as previously described [41]. The arrays were produced by Agilent Technologies, and consisted of 8455 features that represented 5338 predicted protein-encoding open reading frames, tRNA-encoding genes, and rRNA-encoding genes, and 2983 duplicate spots. Cultures of wild-type or the Δ*pdhS2* mutant strain of *A. tumefaciens* strain C58 were grown overnight in ATGN to full turbidity and then sub-cultured 1 : 150 into fresh ATGN for a second overnight growth. The following morning a volume equivalent to 11 ml of OD_600_ 0.6 was prepared for RNA extraction using RNAprotect Bacteria Reagent (QIAGEN, Germantown, MD, USA) following the manufacturer’s protocol. RNA was extracted from these samples using QIAGEN RNA midipreps (QIAGEN, Germantown, MD, USA) following the manufacturer’s protocol. DNA contamination was removed by DNase digestion using the TURBO DNA-free kit (Ambion, Austin, TX, USA) with the incubation time extended to 2 hours. First-strand cDNA synthesis was performed using the Invitrogen SuperScript Indirect Labeling kit, and cDNA was purified on Qiagen QIAquick columns. cDNA was labelled with AlexaFluor 555 and 647 dyes using the Invitrogen SuperScript cDNA Labelling kit, and repurified on QIAquick columns. cDNA was quantified on a NanoDrop spectrophotometer. Hybridization reactions were performed using the Agilent *in situ* Hybridization Kit Plus, boiled for 5 min at 95 °C, applied to the printed arrays and hybridized overnight at 65 °C. Hybridized arrays were washed with Agilent Wash Solutions 1 and 2, rinsed with acetonitrile and incubated in Agilent Stabilization and Drying Solution immediately prior to scanning of the arrays. Three independent biological replicates were performed, with one dye swap. Hybridized arrays were scanned on a GenePix Scanner 4200 in the Center for Genomics and Bioinformatics (CGB) at Indiana University. GenePix software was used to define the borders of hybridized spots, subtract background, measure dye intensity at each spot and calculate the ratio of dye intensities for each spot. Analysis of the scanned images was conducted using the LIMMA package in R/Bioconductor. Background correction of the data was performed using the minimum method [42, 43]. The data were normalized within arrays with the LOESS method, and between arrays with the quantile method. Statistical analysis was performed using linear model fitting and empirical Bayesian analysis by least squares. Genes with significant *P* values (≤0.05) and with log_2_ ratios of ≥0.50 or≤−0.50 (representing a fold change of ±1.4) are reported here. Expression data have been deposited in the Gene Expression Omnibus (GEO) database at the National Center for Biotechnology Information (NCBI) under accession number GSE71267 [44].

β-galactosidase activity was measured using a modified protocol of Miller [45]. Cultures carrying transcriptional reporter plasmids were grown overnight in ATGN and sub-cultured the following morning to an OD_600_ of 0.15. Diluted cultures were grown at 28 °C with aeration until they reached mid-exponential growth. Between 100 and 300 µL of exponential phase culture was mixed with Z buffer (60 mM Na_2_HPO_4_, 40 mM NaH_2_PO_4_, 10 mM KCl, 1 mM MgSO_4_, pH 7.0) to a final volume of 1 ml (volume of culture=*f*) plus two drops of 0.05 % sodium dodecyl sulfate and three drops of CHCl_3_. The amount of culture volume used was calibrated to generate reaction times between 15 min and 2 hours for cultures with activity. Then 0.1 ml of a 4 mg ml^−1^ solution in Z buffer of the colorimetric substrate 2-nitrophenyl β-d-galactopyranoside (ONPG) was added and the time (*t*) required for the solution to turn yellow was recorded. The reaction was stopped by the addition of 1 M Na_2_CO_3_ and the absorbance at 420 nm (A_420_) of each solution was measured. Promoter activity is expressed in Miller units [MU = (1000 ×A_420nm_)/(OD_600nm_× *t* ×*f*)]. Each mutant was tested in three independent experiments containing five technical replicates.

### Global cdGMP measurement

Measurement of cdGMP levels was performed by liquid chromatography–tandem mass spectrometry (LC-MS/MS) on a Quattro Premier XE mass spectrometer coupled with an Acquity Ultra Performance LC system (Waters Corporation), essentially as previously described [46]. Concentrations of cdGMP in cell samples were compared to chemically synthesized cdGMP (Axxora) dissolved in water at concentrations of 250, 125, 62.5, 31.2, 15.6, 7.8, 3.9 and 1.9 nM to generate a calibration curve. *A. tumefaciens* derivatives were grown in ATGN overnight at 28 °C to stationary phase. Culture densities were normalized after the collection of cells by centrifugation and then resuspension in the appropriate volume of ATGN. Cultures were then pelleted by centrifugation and resuspended in ice-cold 250 µL extraction buffer (methanol  : acetonitrile : water, 40  :  40 :  20+0.1 N formic acid) and incubated for 30 min at −20 °C. Resuspensions were transferred to microcentrifuge tubes and pelleted (13 000 r.p.m., 5 min). Then 200 µL of the resulting supernatant was neutralized with 8 µL 15 % NH_4_HCO_3_. Neutralized samples were stored at −20 °C. Prior to mass spectrometric analysis, samples were vacuum-centrifuged to remove the extraction buffer and resuspended in an equal volume of deionized water.

## Results

### Mutational analysis of PdhS2 reveals coordinate regulation through kinase and phosphatase activities

Members of the PdhS family of sensor histidine kinases contain a conserved HATPase_c catalytic domain at their carboxyl termini and an upstream conserved HisKA dimerization/phosphoacceptor domain. Many sensor kinases exhibit bifunctional catalytic activity, alternately acting as a kinase or phosphatase, and *C. crescentus* PleC is one such example [21, 22, 47]. Multiple sequence alignment of the HisKA domain from the *A. tumefaciens* and *C. crescentus* PdhS family kinase homologues reveals a high level of conservation of this domain, including the phospho-accepting histidine residue (H271 of PdhS2) and a threonine residue predicted to be important for phosphatase activity (T275 of PdhS2) (Fig. 1b).

**Fig. 2.**
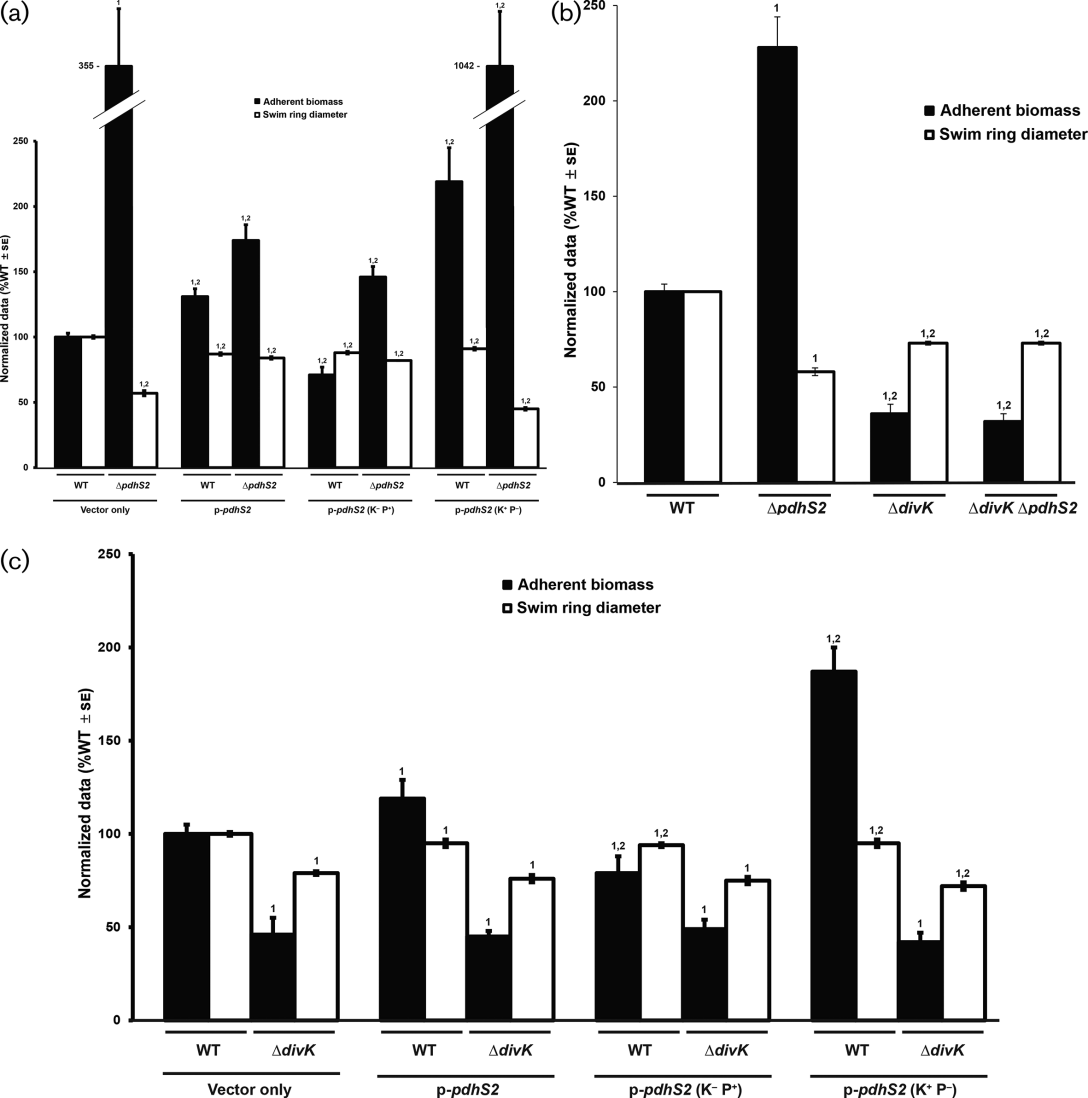
Evaluation of roles for PdhS2 kinase and phosphatase activities and genetic interactions with *divK*. (a) The ability of plasmid-borne wild-type PdhS2 (p-*pdhS2*), the kinase-null allele (p-*pdhS2*, K^−^P^+^), or the phosphatase-null allele (p-*pdhS2,* K^+^P^−^) to complement the Δ*pdhS2* biofilm formation (black bars) and swimming motility (white bars) phenotypes was evaluated using *P_lac_*-driven expression of each allele. Static biofilm formation was measured after 48 h (black bars) and swim ring diameter was measured after 7 days (white bars). Adherent biomass on PVC coverslips was determined by adsorption of crystal violet. Crystal violet was then solubilized and A_600 nm_ values were normalized to culture density (OD_600_). The data are the mean of three independent experiments, each of which contained three technical replicates (*n*=3). Swim ring diameters were measured after single-colony inoculation into low density swim agar and incubation at room temperature. The data are the mean of nine independent experiments (*n*=9). (b) Biofilm formation (black bars) and swimming motility (white bars) were evaluated in the indicated strains. Experiments were performed and data were analysed as described for (a) above. (c) The effect of plasmid-borne wild-type PdhS2 (p-*pdhS2*), the kinase-null allele [p-*pdhS2* (K^−^P^+^)], or the phosphatase-null allele [p-*pdhS2* (K^+^P^−^)] on biofilm formation (black bars) and swimming motility (white bars) when expressed from the *P*_lac_ promoter in the Δ*divK* mutant background was evaluated as in (a) and (b) above. For presentation, all data have been normalized to the wild-type (WT) and they are expressed as %WT ± standard error of the mean (se). 1=*P*<0.05 compared to the wild-type strain or the wild-type strain carrying empty vector. 2=*P*<0.05 compared to the Δ*pdhS2* strain carrying empty vector (a), or compared to the Δ*pdhS2* mutant strain (b), or compared to the Δ*divK* strain carrying empty vector (c). Statistical significance was determined using Student’s *t*-test.

To test the requirement of the conserved phospho-accepting histidine for PdhS2 activity we mutated this residue to alanine (H271A). Ectopic expression of PdhS2^H271A^ (plasmid-borne *P*_lac_-*pdhS2*, predicted kinase-negative, K^−^; phosphatase-positive, P^+^) effectively complemented the attachment and motility phenotypes of the Δ*pdhS2* mutant similar to wild-type *pdhS2* (Fig. 2a). These data indicate that this histidine residue is not crucial for PdhS2 regulation of swimming motility and biofilm formation. When *pdhS2* is expressed ectopically in the wild-type, it causes a slight but significant stimulation of biofilm formation, and the *pdhS2* ^H271A^ (K^−^P^+^) mutation reverses this effect.

The efficient phosphatase activity of many sensor kinases requires a conserved threonine residue roughly one α-helical turn (four residues) downstream of the phospho-accepting histidine residue [48, 49]. We therefore mutated this conserved threonine residue to alanine (Thr275A). In contrast to the PdhS2^H271A^ mutant protein (K^-^P^+^), equivalent ectopic expression of the PdhS2^T275A^ allele (K^+^P^-^) failed to complement the Δ*pdhS2* motility and attachment phenotypes, and in fact exacerbated them (Fig. 2a). When expressed in the wild-type, the PdhS2^T275A^ allele (K^+^P^-^) caused modest stimulation of biofilm formation and slightly decreased motility. A double mutant allele of PdhS2 with both the histidine and threonine residues mutated (K^−^P^−)^ had minimal effect on these phenotypes (Fig. S1). Taken together, these results suggest that it is the balance of kinase and phosphatase activity that dictates PdhS2 control over its targets, with the kinase stimulating biofilm formation and decreasing motility, and the phosphatase activity diminishing biofilm formation and promoting motility. The phosphatase activity, however, appears to play the dominant role under laboratory culture conditions.

### Mutations in *divK* are epistatic to *pdhS2* mutations

Members of the PdhS family of sensor kinases were originally identified based on homology with their namesakes DivJ and PleC of *C. crescentus* (Fig. 1b) [50, 51]. Based on this homology, all PdhS family members are predicted to interact with the single-domain response regulator DivK [51] and also interact with the diguanylate cyclase response regulator PleD. Prior work from our laboratory has shown that both swimming motility and adherent biomass are diminished in the Δ*divK* mutant, implying that DivK activity is required for proper regulation of these phenotypes in *A. tumefaciens* [36]. By contrast, PdhS2 inversely regulates these phenotypes; a Δ*pdhS2* mutant is non-motile but hyperadherent. To determine whether PdhS2 genetically interacts with DivK we constructed a Δ*divK*Δ*pdhS2* mutant and compared swimming motility and biofilm formation in this strain to those in the wild-type and parental single-deletion strains (Fig. 2b). As reported, the loss of either *divK* or *pdhS2* reduced swimming motility as measured by the swim ring diameter on motility agar. Biofilm formation on PVC coverslips in the Δ*divK* mutant was diminished relative to that for the wild-type C58 strain, while for the Δ*pdhS2* mutant it was dramatically increased. The Δ*divK*Δ*pdhS2* mutant was similar to the Δ*divK* mutant in both assays, with no significant difference in the efficiency of either swimming motility or biofilm formation between the two strains. These data support the proposed genetic interaction between *divK* and *pdhS2*, with the *divK* mutation epistatic to *pdhS2* for biofilm formation and swimming motility.

The swim ring diameters of the Δ*divK* and Δ*divK*Δ*pdhS2* mutants were decreased by ~20 % compared to those of the wild-type, whereas there was a ~40 % decrease in the swim ring diameters of the Δ*pdhS2* mutant compared to those of the wild-type, suggesting that the nature of the defect in swimming motility differs between these two classes of mutants and that loss of *divK* partially restores motility in the absence of *pdhS2*. Indeed, it was noted that although both the Δ*divK* and the Δ*pdhS2* single-deletion mutants produce polar flagella, very few Δ*pdhS2* mutant bacteria were observed to be motile under wet-mount microscopy, implying that the swimming defect is due to diminished flagellar activity rather than flagellar assembly [36]. The Δ*divK* mutant, however, was readily observed to be motile under wet-mount microscopy. Similarly, the Δ*divK*Δ*pdhS2* mutant generates polar flagella and its motility is readily observed under wet-mount microscopy. Both the Δ*divK* and Δ*divK*Δ*pdhS2* mutants, and not the Δ*pdhS2* mutant, generate aberrant cell morphologies, including elongated and branched cells (Fig. S2) [36].

Further support for a genetic interaction between PdhS2 and DivK was provided by the plasmid-borne, wild-type PdhS2 expressed ectopically from a *P*_lac_ promoter in backgrounds lacking *divK*. While induced expression of *pdhS2* rescues swimming motility and returns biofilm formation closer to wild-type levels in the Δ*pdhS2* mutant, albeit incompletely (Fig. 2a), plasmid-borne provision of PdhS2 in either the Δ*divK* or Δ*divK*Δ*pdhS2* mutant has no significant effect on either biofilm formation or swimming motility (Fig. 2c). Expression of either the predicted kinase-null or the predicted phosphatase-null allele of PdhS2 in the Δ*divK* background similarly has no effect on biofilm formation or swimming motility (Fig. 2c).

### Kinase-biased allele of CckA does not suppress *pdhS2* phenotypes

**Fig. 3.**
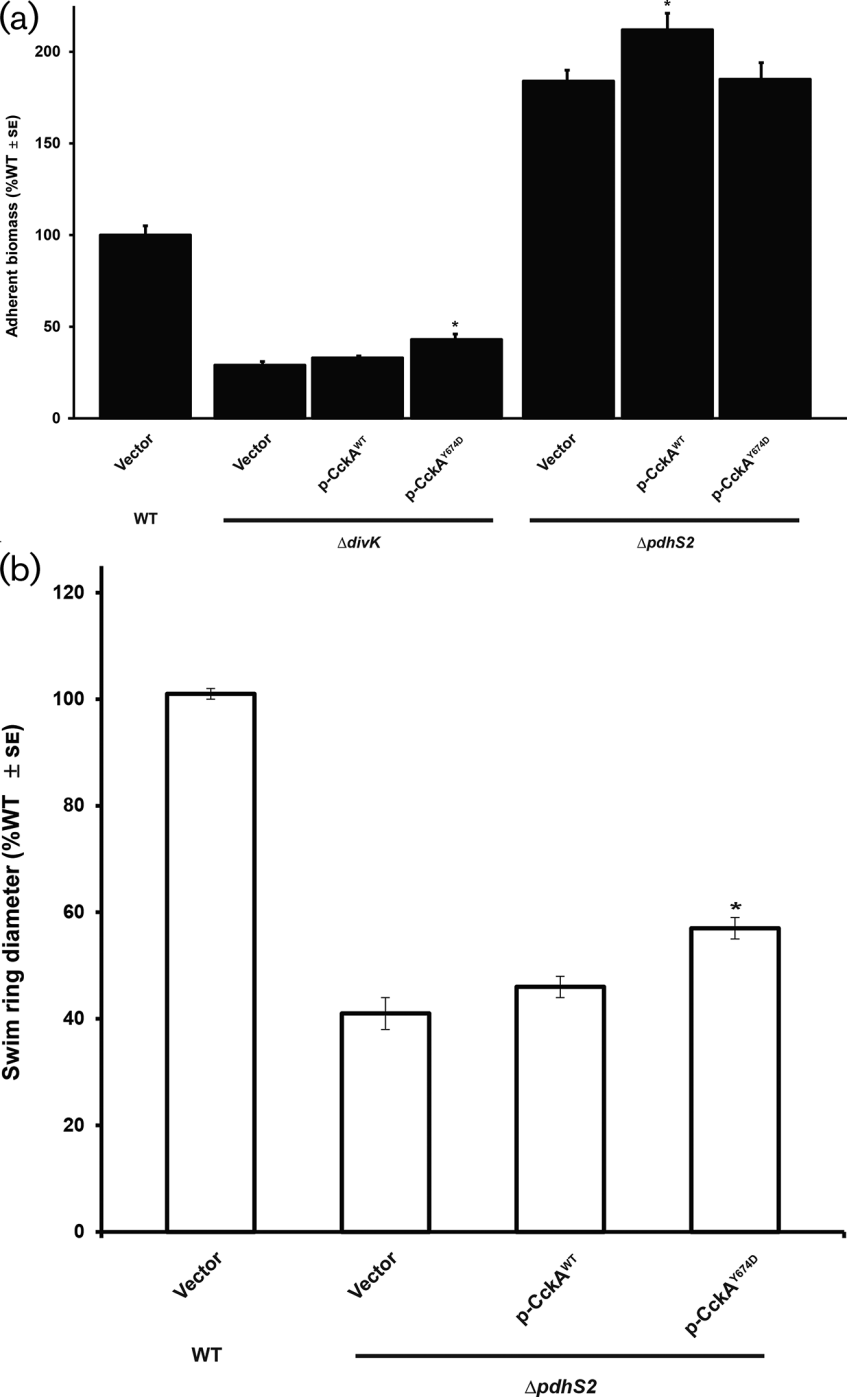
A kinase-locked allele of CckA fails to suppress the PdhS2-dependent biofilm and motility phenotypes. (a) Biofilm formation was evaluated in the indicated strains as described in Fig. 2. *, *P*<0.05 compared to background strain carrying vector alone. Statistical significance was determined using Student’s *t*-test. (b) Swimming motility was evaluated in the indicated strains as described in Fig. 2. *, *P*<0.001 compared to background strain carrying vector alone. Statistical significance was determined using Student’s *t*-test.

One key indirect PdhS target among multiple bacterial taxa is the hybrid histidine kinase CckA. CckA exhibits dynamic regulation that is dependent upon both the phosphorylation status of DivK and local levels of cdGMP [33]. CckA ultimately serves as either a source or a sink for CtrA phosphorylation through a phosphorelay that includes the histidine phosphotransferase ChpT [30]. Previously we identified a mutation in CckA that results in a kinase-biased allele that is insensitive to regulation by cdGMP, CckA^Y674D^ [33, 36]. Expression of this allele in the Δ*pleC* background suppressed the swimming motility defect of the Δ*pleC* strain but had no effect on swimming motility in the Δ*divK* background [36]. These results were consistent with PleC activity proceeding through DivK and PleD and with DivK negatively regulating CckA kinase activity. To determine the role played by CckA in PdhS2-dependent phenotypes, we first evaluated swimming motility in a Δ*pleC* Δ*pleD* background to verify the role played by PleD in this pathway in *A. tumefaciens *. Loss of PleD activity restored swimming motility in a strain also lacking PleC (Fig. S3). These data are consistent with PleC’s role in *A. tumefaciens* mirroring that seen in *C. crescentus *: PleC negatively regulates PleD activity, thereby reducing local levels of cdGMP available to bias CckA towards phosphatase activity. We reasoned that if PdhS2 similarly functioned through DivK, PleD and CckA, expression of CckA^Y674D^ in the Δ*pdhS2* background would suppress the motility and biofilm phenotypes of this strain. However, induced expression of wild-type CckA or the CckA^Y674D^ allele only marginally affected these phenotypes (Fig. 3). These observations suggest that PdhS2 functions differently than PleC, either by interacting with DivK and PleD at a different time in the cell cycle or by interacting with additional regulatory targets.

### Expression of predicted CtrA-dependent promoters

The known architecture of the DivK–CtrA pathway in other alphaproteobacteria predicts that PdhS2 impacts on developmental phenotypes through the transcriptional regulator CtrA. In *C. crescentus *, CtrA is known to directly regulate at least 55 operons, acting as either an activator or repressor of transcription, and to control DNA replication [22, 27]. *A. tumefaciens* CtrA is predicted to act similarly, binding to DNA in a phosphorylation-dependent manner and regulating DNA replication and transcription. *A. tumefaciens* CtrA is 84 % identical to *C. crescentus* CtrA at the amino acid level and purified *C. crescentus* CtrA binds to a site upstream of the *A. tumefaciens ccrM* gene [52]. Furthermore, computational analysis of multiple alphaproteobacterial genomes uncovered numerous cell cycle-regulated genes preceded by a consensus CtrA-binding site [53]. We therefore evaluated CtrA activity by examining the transcription of several known and predicted CtrA-dependent promoters from both *C. crescentus* and *A. tumefaciens* in wild-type, Δ*pdhS2* and Δ*divK A. tumefaciens* strain backgrounds. The *ccrM*, *ctrA* and *pilA* promoters from *C. crescentus* were chosen to represent CtrA-activated promoters likely to be similarly regulated in *A. tumefaciens* [13, 14, 54, 55]. In the Δ*pdhS2* background, expression levels from both the *ctrA* and *pilA* promoters from *C. crescentus* were significantly reduced. while transcription from the *C. crescentus ccrM* promoter was unchanged (Table 1). In the *A. tumefaciens* Δ*divK* background the *C. crescentus ccrM* and *ctrA* promoters exhibited increased activity, while the *pilA* promoter was unchanged (Table 1). These data are consistent with *A. tumefaciens* CtrA regulating the transcription of known CtrA-dependent promoters, and with PdhS2 and DivK differentially regulating CtrA activity in *A. tumefaciens *.

**Table 1.** Promoter activity of selected known and predicted CtrA-dependent promoters*

**Strain**	**Promoter source organism**	**Promoter**	**Activity (%WT±se)**
WT	* C. crescentus *	*ccrM*	100±1
		*ctrA*	100±2
		*pilA*	100±1
	* A. tumefaciens *	*ccrM*	100±1
		*ctrA*	101±1
		*pdhS1*	100±1
		*dgcB*	100±1
		dgcB*†	278±15‡, §
		Atu3318	101±1
		Atu3318*	763±25‡, §
Δ*pdhS2*	* C. crescentus *	*ccrM*	109±12
		*ctrA*	82±4§
		*pilA*	85±2§
	* A. tumefaciens *	*ccrM*	106±2§
		*ctrA*	84±3§
		*pdhS1*	119±3§
		*dgcB*	247±10§
		dgcB*	614±23‡, §, ||
		Atu3318	295±19§
		Atu3318*	897±41‡, §, ||
Δ*divK*	* C. crescentus *	*ccrM*	140±6§
		*ctrA*	132±4§
		*pilA*	99±3
	* A. tumefaciens *	*ccrM*	116±3§
		*ctrA*	117±2§
		*pdhS1*	90±1§
		*dgcB*	80±4§
		dgcB*	199±9‡, §, ||
		Atu3318	75±3§
		Atu3318*	724±40‡, §

*Promoter activity was measured as the β-galactosidase activity of cell lysates from strains carrying either transcriptional (for *C. crescentus* promoters) or translational (for *A. tumefaciens* promoters) fusions of the indicated promoter regions to the *E. coli lacZ* gene. Activity for each promoter was normalized to activity in lysates from wild-type cells carrying the identical (wild-type) promoter. *n*=9.

†dgcB* and Atu3318* indicate mutated promoter regions where the predicted CtrA-binding motif has been switched from 5′-TTAA-3′ to 5′-AATT-3′.

‡*P*<0.05 compared to same strain carrying wild-type promoter construct using Student’s *t*-test.

§*P* <0.05 compared to WT strain carrying wild-type promoter construct using Student’s *t*-test.

||*P* <0.05 compared to WT strain carrying mutant promoter construct using Student’s *t*-test.

For *A. tumefaciens* the *ccrM* promoter is the only promoter for which prior experimental data suggest CtrA-dependent regulation, thus this promoter was selected for analysis [52]. In addition to *ccrM*, putative *A. tumefaciens* promoters for *ctrA* and *pdhS1* were selected for analysis based on the presence of at least one predicted CtrA-binding site, as well as hypothesized cell cycle regulation of these loci. Transcriptional activity from the *A. tumefaciens ctrA* and *pdhS1* promoter constructs showed inverse regulation in the Δ*pdhS2* and Δ*divK* backgrounds, with expression decreased for the *ctrA* promoter and increased for the *pdhS1* promoter in the Δ*pdhS2* mutant, and exactly reversed in the Δ*divK* mutant. Although the absence of *pdhS2* had little effect on the *A. tumefaciens ccrM* promoter, transcription from this promoter was significantly increased in the Δ*divK* background (Table 1). These data are congruent with the above data for *C. crescentus* CtrA-dependent promoters and further support CtrA regulation of cell cycle-responsive genes in *A. tumefaciens *.

### Global transcriptional analysis of PdhS2 activity

To determine the effect of PdhS2 activity on the *A. tumefaciens* transcriptome we used whole-genome microarrays. Gene expression was compared between wild-type and Δ*pdhS2* strains grown to exponential phase in minimal media. Of 5338 unique loci represented on the arrays, 39 genes were differentially regulated above our statistical cut-offs (*P* values≤0.05; log_2_ ratios of ≥±0.50; Table 2). Of these, 24 genes were significantly upregulated, indicating negative regulation by PdhS2. The upregulated genes included *dgcB*, which had previously been shown to contribute to elevated biofilm formation in hyperadherent *A. tumefaciens* mutants disrupted in the motility regulators VisN and VisR [56]. Also upregulated was Atu3318, encoding a LuxR-type transcription factor, that, similar to *dgcB*, was previously identified by its elevated levels in *visNR* null mutants. The downregulated genes in the *pdhS2* mutant included six succinoglycan biosynthetic genes, which is consistent with our previous results showing positive regulation of succinoglycan production by PdhS2 [36].

**Table 2.** Differentially regulated genes in the absence of pdhS2*

**Locus**	**Gene**	**Product†**	**log_2_ FC**	**CtrA-binding site (+/−)‡**	**CtrA half-site (+/−)‡**	**Present in other PdhS arrays?§**
Atu0461	−	*Phage tail protein/type VI secretion system component*	2.42	(+) Atu8128||	+	No
Atu0227	−	tRNA-Leu	2.15	−	+	No
Atu5167	*avhB6*	Type IV secretion protein	2.01	−	+	No
Atu2490	*asd*	Aspartate semialdehyde dehydrogenase	1.93	−	+	No
Atu3318	−	LuxR family transcriptional regulator	1.84	+	+	No
Atu1471	*rluC*	Ribosomal large subunit pseudouridine synthase C	1.84	−	+	No
Atu3755	*purK*	Phosphoribosylaminoimidazole carboxylase ATPase subunit	1.70	−	+	No
Atu3606	*ftsE*	Cell division ATP-binding protein	1.63	+	+	No
Atu1791	−	ABC transporter, membrane spanning protein (sugar)	1.55	−	+	No
Atu3572	−	XRE family transcriptional regulator	1.52	+	+	Yes
Atu2217	−	*Hypothetical protein*	1.52	−	+	No
Atu5119	*phoB*	Two-component response regulator	1.48	−	−	No
Atu3031	−	*Hypothetical protein*	1.46	−	−	No
Atu1886	−	*DNA glycosylase*	1.41	−	−	No
Atu1964	−	tRNA-Trp	1.40	+	+	No
Atu1301	−	*Nudix hydrolase*	1.39	−	+	No
Atu3610	−	Cation transporter	1.34	−	−	No
Atu6048	−	*RNA helicase*	1.32	−	+	No
Atu1691	*dgcB*	GGDEF family protein	1.32	+	+	No
Atu1887	*exoI*	Succinoglycan biosynthesis protein	1.30	+	+	No
Atu1134	−	*Lysyl-phosphatidylglycerol synthase*	1.30	−	−	No
Atu2665	−	MarR family transcriptional regulator	1.27	−	+	No
Atu2204	−	*Hypothetical protein*	1.25	−	+	No
Atu0540	−	*hypothetical protein*	1.25	−	−	No
Atu4856	−	Nucleotidyltransferase	−1.21	−	−	No
Atu4347	*tae*	Type VI secreted effector	−1.23	(+) Atu4344	−	No
Atu4055	*exoK*	Endo-1,3–1,4-beta-glycanase	−1.28	(+) Atu4056	+	Yes
Atu4345	*tssD*	Type VI secretion needle tube protein	−1.29	(+) Atu4344	+	No
Atu5091	*rcdB*	Curdlan synthesis protein	−1.32	+	+	No
Atu4357	−	*Transglutaminase-like Cys protease*	−1.34	−	+	Yes
Atu0343	*barA*	Two-component sensor kinase/response regulator hybrid	−1.37	−	−	No
Atu4053	*exoA*	Succinoglycan biosynthesis protein	−1.42	(+) Atu4056	+	Yes
Atu4056	*exoH*	Succinoglycan biosynthesis protein	−1.42	+	+	Yes
Atu3564	*exsH*	Endo-1,3–1,4-beta-glycanase	−1.46	−	−	No
Atu3541	−	*Transglutaminase-like Cys protease*	−1.51	−	+	No
Atu4627	−	*Hypothetical protein*	−1.56	−	−	No
Atu4049	*exoP*	Exopolysaccharide polymerization/transport protein	−1.64	(+) Atu4056	+	No
Atu1469	−	Hypothetical protein	−1.80	+	+	No
Atu4050	*exoN*	UTP-glucose-1-phosphate uridylyltransferase	−1.82	(+) Atu4056	+	Yes

*Gene expression was compared between the wild-type and Δ*pdhS2* strains using whole-genome microarrays. Genes were defined as differentially regulated when the following conditions were met: log_2_ FC ≥0.50 OR log_2_ FC ≤−0.50, and *P*<0.050, and Q<0.10.

†Predicted functions of hypothetical proteins, if available, are italicized.

‡Intergenic regions 500 nt upstream and 100 nt downstream of start codon for each gene (or operon, if applicable) were scanned for possible CtrA-binding sites, as described in the text.

§Results were compared with previously published *S. meliloti cbrA* and *divJ* microarrays and *C. crescentus divJ* and *pleC* microarrays, as described in the text.

||(+) with Atu number indicates that the putative CtrA box is located in the first gene of a predicted operon.

To determine whether any of these 39 genes were putatively regulated by CtrA we scanned a sequence window from 500 bp upstream of the start codon to 100 bp into the coding sequence for plausible CtrA-binding sites. CtrA-binding sites were defined using the conserved alphaproteobacterial CtrA recognition sequence 5′-TTAANNNNNNGTTAAC-3′ [52, 53]. Sequences containing seven or more of the conserved nucleotides in this motif were deemed plausible candidates. Using these parameters, 15 differentially transcribed loci are expressed from promoters (some from upstream genes in the operon) with putative CtrA-binding sites and are thus putatively directly regulated by CtrA, including *dgcB* and Atu3318, as well as all 5 of the downregulated succinoglycan biosynthetic genes (Fig. S4; Table 2). We also identified numerous CtrA half-sites containing the sequence 5′-TTAA-3′. In *C. crescentus* CtrA has been shown to bind to such half-motifs, resulting in transcriptional effects [57]. Twenty-eight promoters contained at least one CtrA half-site (Table 2).

To extend our microarray results we measured transcription of translational fusions to β-galactosidase for *dgcB* and Atu3318 in wild-type, Δ*pdhS2* and Δ*divK* strain backgrounds (Table 1). In both cases, β-galactosidase activity increased in the Δ*pdhS2* mutant, corroborating the microarray results. Furthermore, activity decreased from each promoter fusion in the Δ*divK* background, supporting inverse regulation by PdhS2 and DivK at these promoters. Finally, to confirm a role for CtrA in transcriptional regulation by PdhS2 and DivK, we mutated the first four nucleotides of the predicted CtrA-binding motif (−52 from the start codon, TTAA to AATT) in the *dgcB* promoter region and evaluated transcription from this promoter fused to β-galactosidase as above. In all cases, activity from this promoter was derepressed, supporting CtrA-dependent regulation (Table 1). Similar mutational analysis of the predicted CtrA-binding site in the Atu3318 promoter (−109 from the start codon) resulted in elevated expression in wild-type, Δ*divK* and Δ*pdhS2* backgrounds, consistent with CtrA repressing transcription from this promoter. Overall, these results are consistent with PdhS2 impacting on the motile cell developmental program through CtrA-dependent transcriptional control.

### PdhS2 activity intersects with cyclic-di-GMP pools

**Fig. 4.**
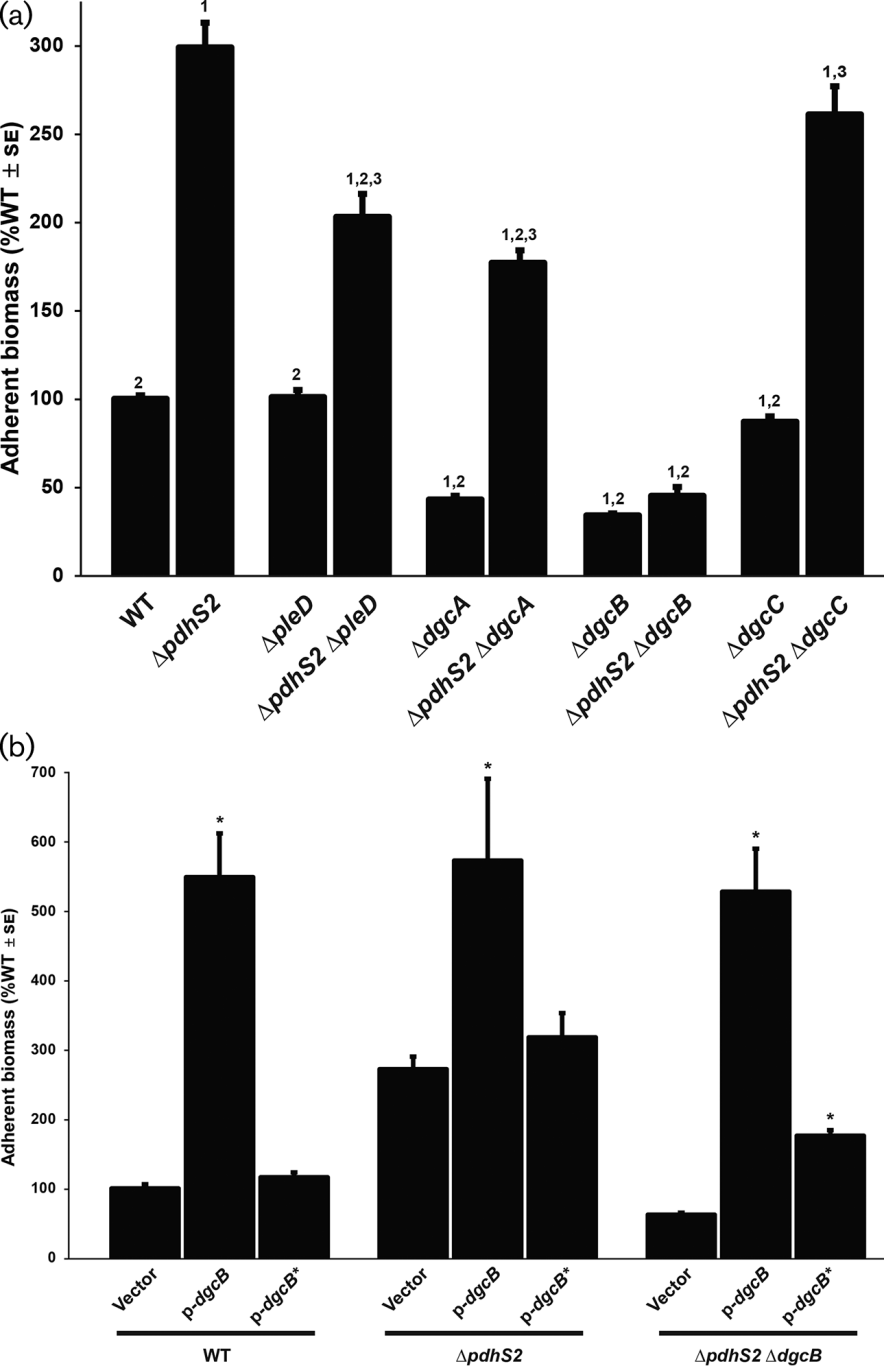
PdhS2 intersects with the activity of multiple diguanylate cyclases. (a) Biofilm formation was quantified for the wild-type (WT) and indicated mutant strains as described in Fig. 2. PleD, DgcA and DgcB have demonstrated *in vivo* diguanylate cyclase enzymatic activity. Thus far, the conditions under which DgcC is active have yet to be identified. *P*<0.05 compared to WT (1), Δ*pdhS2* (2), or the corresponding diguanylate cyclase mutant (3). (b) The effect on biofilm formation of plasmid-borne expression of wild-type *dgcB* (p-*dgcB*) or a catalytic mutant allele of *dgcB* (p-dgcB*) was evaluated. Expression of each *dgcB* allele was driven by the *P_lac_* promoter. Biofilm formation was evaluated as described in Fig. 2. *, *P*<0.05 compared to vector alone.

Increased attachment coupled with decreased motility is a hallmark of regulation by the second messenger cdGMP. In *C. crescentus *, DivJ and PleC positively regulate, via phosphorylation, a second response regulator, the diguanylate cyclase PleD, as well as DivK [22]. In *C. crescentus* and *A. tumefaciens *, the *divK* and *pleD* coding sequences form one operon and transcriptional regulation of both genes is linked. Since several PdhS kinases in these systems are predicted to interact with both DivK and PleD, we analysed the effect of loss of PleD activity in the Δ*pdhS2* background. As reported previously, deletion of *A. tumefaciens pleD* alone has only modest effects on swimming motility and adherent biomass [36]. Loss of *pleD* in the Δ*pdhS2* background had a minimal effect on swimming motility (Fig. S5). Biofilm formation, however, was reduced by approximately 30 %, indicating that PleD contributes to the increased attachment phenotype of the Δ*pdhS2* mutant (Fig. 4a).

**Fig. 5.**
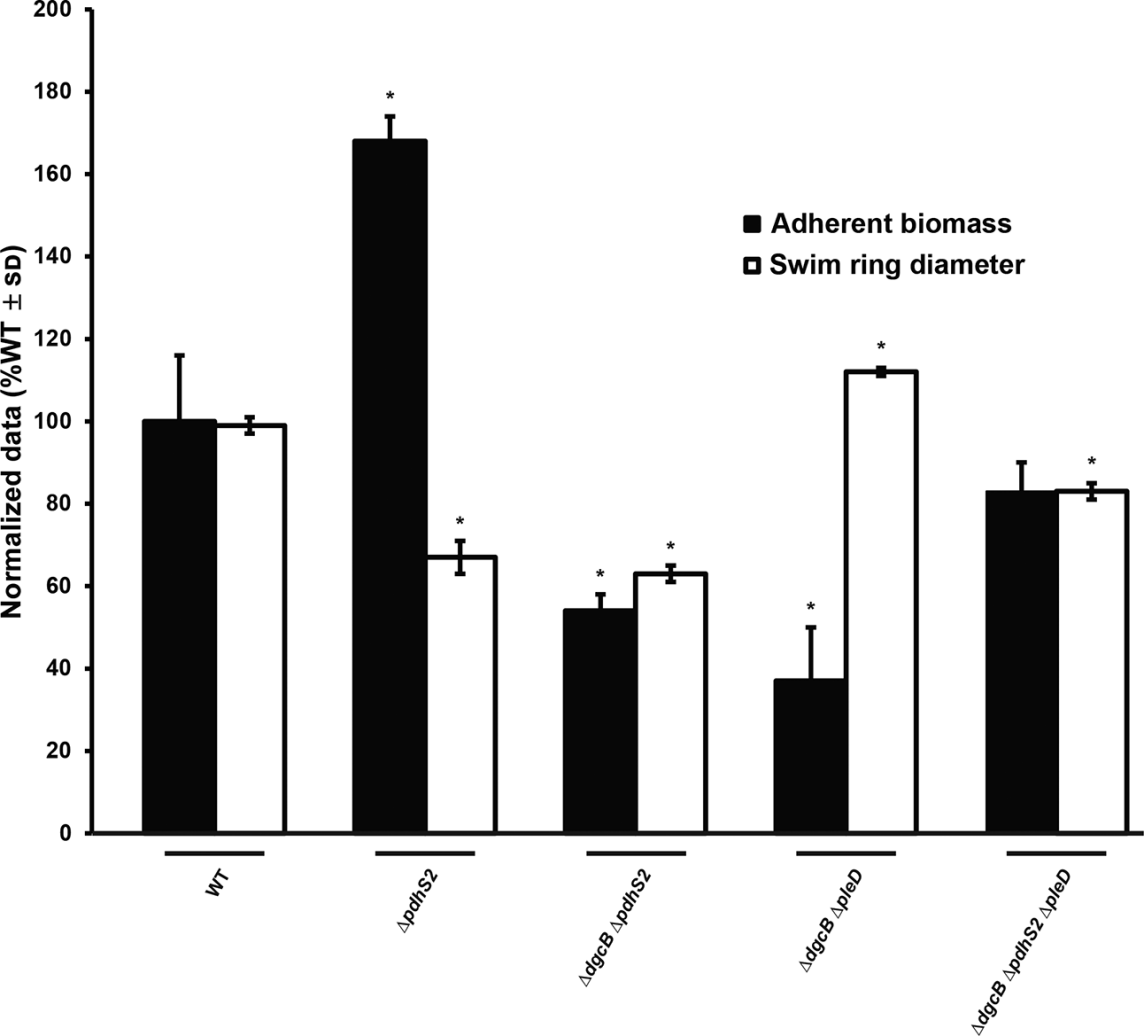
Loss of *pdhS2* enhances biofilm formation in the absence of both *dgcB* and *pleD*. Biofilm formation and swimming motility was evaluated in the wild-type (WT) and indicated mutant strains as described in Fig. 2. *, *P*<0.05 compared to the wild-type background. Statistical significance was determined using Student’s *t*-test.

PleD is a GGDEF motif containing diguanylate cyclase (DGC), and thus it is likely that the attachment phenotype of the Δ*pdhS2* mutant requires increased levels of cdGMP. Earlier work from our laboratory identified three additional DGCs that are relevant to attachment and biofilm formation: DgcA, DgcB and DgcC [56]. As seen in wild-type C58, deletion of *dgcA* or *dgcB* in the Δ*pdhS2* background significantly decreased attachment and biofilm formation, whereas loss of *dgcC* did not (Fig. 4a). These data suggest that increased biofilm formation by the Δ*pdhS2* mutant is dependent on cdGMP pools, generated through PleD, DgcA, or DgcB. Loss of *dgcB* largely abolishes the increased biofilm formation of the Δ*pdhS2* mutant. We also compared biofilm formation in Δ*dgcB*Δ*pleD* and Δ*pdhS2*Δ*dgcB*Δ*pleD* mutant backgrounds. In the absence of both DgcB and PleD, biofilm formation was enhanced by loss of PdhS2 (Fig. 5). These data suggest that the increased biofilm formation of a *pdhS2* mutant is dependent on a cdGMP pool that is predominantly due to DgcB, but that is also under the cumulative influence of multiple DGC enzymes. Swimming motility was equivalent in either wild-type C58 or Δ*pdhS2* backgrounds in combination with mutations in *pleD*, *dgcA*, *dgcB*, or *dgcC* (Figs 5 and S5).

Previously we found that mutants lacking either *dgcA*, *dgcB*, or *dgcC* show insignificant differences in total cytoplasmic levels of cdGMP [56]. Nonetheless, loss of either *dgcA* or *dgcB*, or mutation of the GGDEF catalytic site of either enzyme, significantly reduced biofilm formation [56], implicating these enzymes in controlling the pool of cdGMP and thereby affecting attachment. We compared cytoplasmic cdGMP levels for wild-type C58, the Δ*pdhS2* mutant and the Δ*pdhS2*Δ*dgcB* mutant strain and found these levels to be low, with no significant change between mutants (Fig. S6). To verify that the DGC activity is responsible for the increased biofilm formation in the Δ*pdhS2* background we expressed an allele of *dgcB* with a mutation in its GGDEF catalytic motif (GGAAF; dgcB*) that abrogates cdGMP formation and that fails to complement a Δ*dgcB* mutant for either cdGMP formation or attachment phenotypes [56]. Plasmid-borne expression of wild-type *dgcB* (*P_lac_-dgcB*) results in a massive increase in attachment and biofilm formation in either the wild-type C58 or Δ*pdhS2* background (Fig. 4b). Expression of dgcB* from a *P_lac_* promoter, however, did not increase biofilm formation in either background. In the Δ*pdhS2*Δ*dgcB* mutant background expression of wild-type *dgcB* increased biofilm formation to the same degree that was seen in the wild-type and Δ*pdhS2* mutant. Expression of the mutant dgcB* allele in the Δ*pdhS2*Δ*dgcB* mutant did appear to modestly affect biofilm formation, although far less than with the wild-type *dgcB* allele. Swimming motility was modestly but significantly reduced when the wild-type *dgcB* allele was provided *in trans* and this was abolished by the dgcB* mutation (Fig. S7). Taken together, our data are consistent with PdhS2-dependent biofilm formation and, to a lesser extent, swimming motility, being mediated at least in part by cdGMP levels.

### Increased attachment in a *pdhS2* mutant requires the UPP polysaccharide

**Fig. 6.**
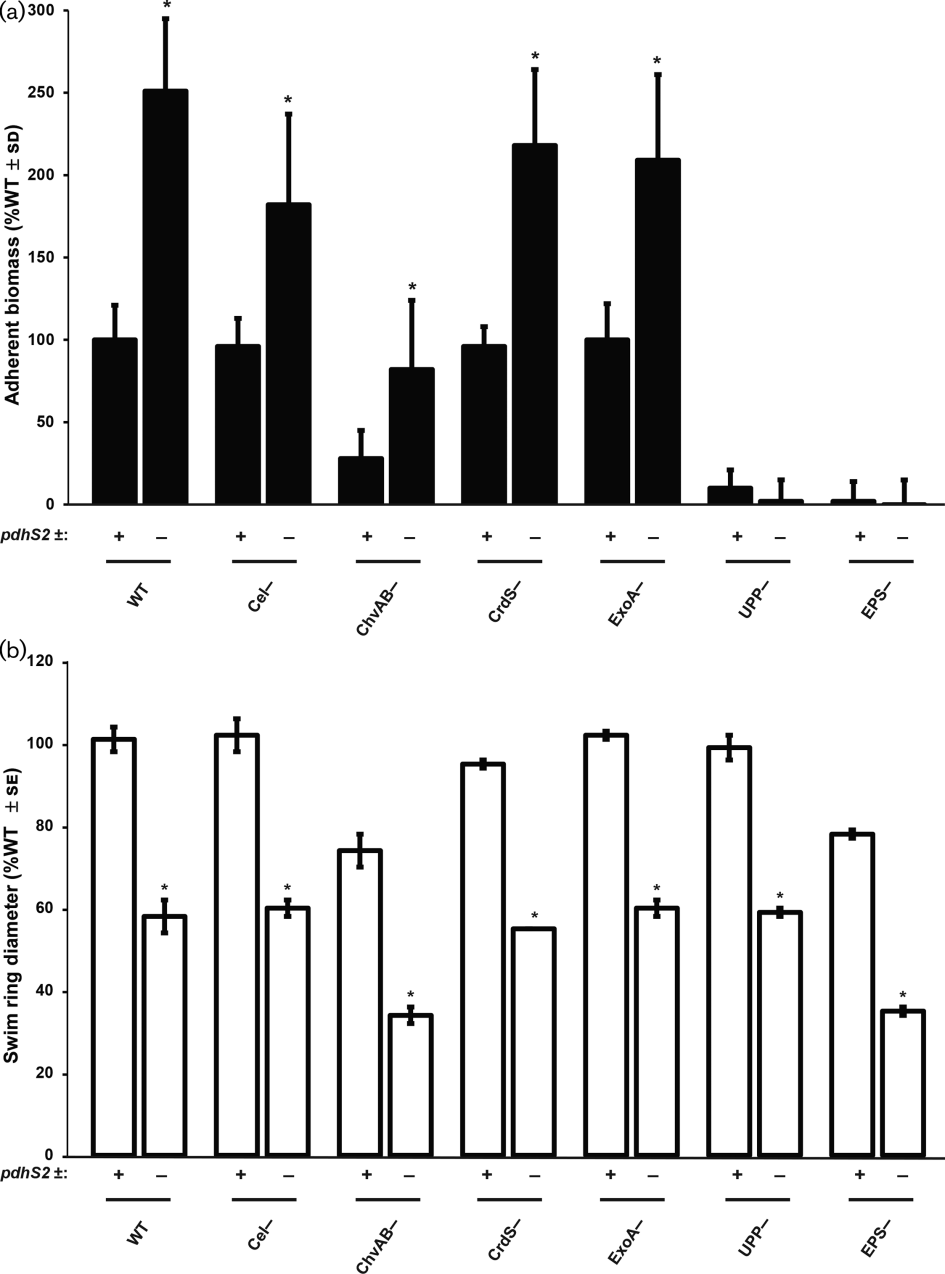
The unipolar polysaccharide is required for PdhS2-dependent biofilm formation. (a) Biofilm formation was evaluated in the presence (+) or absence (−) of *pdhS2* in combination with mutants deficient in production of the indicated polysaccharides. WT=wild type, Cel^−^=cellulose mutant, ChvAB^−^=cyclic-β-glucan mutant, CrdS^−^=curdlan mutant, ExoA^−^=succinoglycan mutant, UPP^−^=unipolar polysaccharide mutant, EPS^−^=mutant lacking all of the above polysaccharides. (b) Swimming motility was evaluated in the same strains as in (a). *, *P*<0.05 compared to the PdhS2+ background strain. Statistical significance was determined using Student’s *t*-test.

We have previously reported that PleD-stimulated attachment was due to increased levels of the unipolar polysaccharide (UPP) and cellulose [56]. In addition to UPP and cellulose, *A. tumefaciens* produces at least three other exopolysaccharides: succinoglycan, cyclic β−1,2 glucan and β−1,3 glucan (curdlan), as well as outer membrane-associated lipopolysaccharide (LPS) [58]. Of these, only LPS is essential for *A. tumefaciens* growth [35]. The Δ*pdhS2* strain was tested for the impact of each of the non-essential exopolysaccharides on biofilm formation and swimming motility. The *upp* mutation completely abolished attachment in both the wild-type and the *pdhS2* mutant (Fig. 6a). The *chvAB* mutant, defective in synthesis of cyclic β−1,2 glucan and known to have pleiotropic effects [59], was diminished in adherence overall, but was still elevated by the *pdhS2* mutation. None of the other exopolysaccharide pathways affected adherence in either background. The decreased swimming phenotype of the *pdhS2* mutant was not significantly altered for any of the exopolysaccharide mutants (Fig. 6b). These results indicate that biofilm formation in the Δ*pdhS2* strain is dependent primarily on UPP production and that the motility phenotype of the Δ*pdhS2* mutant is not dependent on any of the known exopolysaccharides.

## Discussion

### PdhS2 regulates attachment and motility predominantly by its phosphatase activity and through CtrA

Regulation of the developmental program of many alphaproteobacteria centres on the global transcriptional regulator CtrA [53, 60–62]. CtrA activity is controlled, indirectly, through a series of phosphotransfer reactions that are dependent on one or more PdhS-type histidine kinases. Here we show that PdhS2, one of at least four PdhS family kinases from *A. tumefaciens *, regulates motility and attachment, most likely through fine-tuning of CtrA activity, thereby impacting on the CtrA regulon. Our data support altered CtrA activity as being responsible for many of the *pdhS2*-dependent transcriptional responses, and through these motility and attachment. A significant fraction of the differentially expressed genes have presumptive CtrA-binding sites in their upstream regions. We demonstrate that null mutations of the single domain response regulator *divK* are epistatic to *pdhS2* mutations in *A. tumefaciens *. Mutation of specific DGCs, including PleD, also reverse the phenotypes of *pdhS2* mutants, suggesting that elevated attachment and decreased motility are mediated through cdGMP pools. The predicted phosphatase activity of PdhS2 is predominantly responsible for its function during laboratory culture growth. It is worth noting, however, that strains bearing the phosphatase-null and kinase-null alleles of PdhS2 do not phenocopy one another or the Δ*pdhS2* mutant strains, supporting distinct roles for both enzymatic activities. This is in contrast to mutant alleles of *pleC* in *C. crescentus* [63–65]. Fine-scale determination of the timing, specificity and regulation of both phosphatase and kinase activities await further experimentation. Similarly, biochemical confirmation of both kinase and phosphatase activities, as well as identification of direct targets for PdhS2, will require the generation of active, purified protein and detailed phosphotransfer profiling of the entire PdhS sensor kinase suite from *A. tumefaciens* [66]. It should be noted that these experiments use overexpression of PdhS2 alleles, and PdhS2 activity relative to the cell cycle may not represent wild-type conditions. A more detailed temporal analysis of PdhS2 regulatory effects awaits the construction of single-copy, native *P_pdhS2_*-driven expression of each allele.

### PdhS2 influences cdGMP-dependent phenotypes

The increased biofilm formation and diminished motility in a *pdhS2* mutant is most similar to the inverse regulation frequently observed for increasing internal pools of cdGMP [67]. Indeed, our data demonstrate a strong dependence on specific diguanylate cyclases for mediating the Δ*pdhS2* hyperadherent phenotype, and transcription analysis demonstrates that *dgcB* expression is elevated in this mutant. Although measurements of the cytoplasmic cdGMP levels suggest that they remain low overall in the *pdhS2* mutant, it is clear that mutations in *dgcB* strongly reverse the effects of the *pdhS2* mutation on attachment and that, to a lesser extent, mutations in *dgcA* and *pleD* can diminish them. This suggests that increased cdGMP synthesis via these enzymes may impart the effect on UPP-dependent attachment. Although DgcB seems to have the dominant effect, it is plausible that PdhS2 may affect PleD DGC activity through the phosphorylation state of its receiver domain, similar to other PleD homologues [22, 68, 69]. In *A. tumefaciens* PleD has only modest effects on motility and attachment, and does not contribute significantly to cell cycle control [36]. Neither DgcB nor DgcA is a response regulator, and it is more likely that at least for *dgcB,* its elevated expression is the mechanism through which CtrA functions. Recent studies have revealed that the *C. crescentus* orthologue of *A. tumefaciens* DgcB regulates holdfast synthesis in response to changes in flagellar rotation [70]. Thus, changes in *dgcB* levels due to mutation of *pdhS2* may be also be affecting the motile to sessile transition in *A. tumefaciens *. Interestingly, as shown for *C. crescentus* and *A. tumefaciens *, elevated cdGMP allosterically switches the bifunctional hybrid histidine kinase CckA from the kinase to the phosphatase mode, thereby downregulating CtrA phosphorylation and DNA-binding activity [32, 33]. Thus effects on local cdGMP levels can feed back on the CtrA pathway, reinforcing decreases in CtrA ~P that would be coincident with increased cdGMP.

The phenotypes regulated by PdhS2 mirror those regulated by the master motility regulators VisR and VisN [56]. Loss of either *visN* or *visR* results in abolishment of motility and a dramatic increase in attachment that is dependent on cdGMP production and the UPP adhesin. However, the motility defect in *visNR* mutants is predominantly transcriptional, as expression of all of the flagellar genes is dramatically decreased. In contrast, none of the flagellar genes are differentially regulated in the *pdhS2* mutant as measured in our microarray data, and flagella are assembled but decreased in activity. The increased attachment in *visNR* mutants is, however, due to elevated *dgcB* expression and also requires *dgcA*, through increased cdGMP and elevation of UPP and cellulose biosynthesis [56]. Interestingly, other target genes that are derepressed in *pdhS2* mutants are also among the small fraction of the genes that are increased in a *visR* mutant, such as Atu3318. Given the presence of CtrA boxes in their upstream sequences, this may suggest a common underlying mechanism. Interestingly, mutation of *dgcB* or the other DGCs does not enhance the dramatically impeded motility of the *pdhS2* mutant. This suggests that the loss of motility in the *pdhS2* mutant is not primarily due to elevated cdGMP levels. In many systems, CtrA regulates motility directly, often through flagellar gene expression [71, 72]. In fact, plasmid-borne expression of the *A. tumefaciens* CtrA in a *ctrA*-null mutant of the marine alphaproteobacterium *Ruegeria* sp. KLH11 (*ctrA* is not essential in this taxon) effectively reverses its non-motile phenotype [73], which is indicative of its positive impact on the motility of this bacterium.

### Segregation of antagonistic signalling activity promotes asymmetric development

The asymmetric division of *A. tumefaciens* and other alphaproteobacteria, producing two genetically identical but phenotypically distinct daughter cells, requires well-coordinated regulation of two developmental programs. The mother cell remains in a terminally differentiated state, proceeding through distinct synthesis (S) and growth (G1/G2) phases of the cell cycle [58, 61]. During the G1/G2 phase the cell elongates into a predivisional cell and establishes a functional asymmetry between its two cellular poles by differential localization of antagonistic homologues of the PdhS kinases. At least one PdhS kinase localizes to the old pole; DivJ in *C. crescentus *, DivJ and PdhS1 in *A. tumefaciens* [74] (Figs 1a and S8b), PdhS in *B. abortus* and CbrA in *Sinorhizobium meliloti* [21, 50, 74]. From this position these kinases can act to phosphorylate targets such as DivK and PleD, indirectly inactivating CtrA (as reported for *C. crescentus *). At the opposite pole, at least one PdhS kinase, PleC in *C. crescentus *, and PdhS2 in *A. tumefaciens* [74] (Figs 1a and S8a), localizes and acts primarily through its phosphatase activity to dephosphorylate targets, ultimately promoting CtrA stability and activity. Upon cytokinesis, then, the motile daughter cell is released in a G1/G2 growth phase with high levels of CtrA activity establishing a distinct transcriptional program and limiting DNA replication.

Our data are consistent with PdhS2 acting in the motile daughter cell to prevent premature activation of cell attachment processes, as well as to promote motility. PdhS2 dynamically localizes to the new pole of *A. tumefaciens* cells following cytokinesis, while DivJ, another PdhS-type kinase, localizes to the old pole of each cell. We propose that together the antagonistic activities of DivJ and PdhS2 (and perhaps additional PdhS homologues), coupled with their distinct localization patterns, generate a spatiotemporal gradient of phospho-CtrA, thus differentially regulating the developmental program of *A. tumefaciens* (Fig. 1a). Localized synthesis and degradation of cdGMP contributes to this regulatory gradient. Prior to and after cytokinesis, daughter cells would have PdhS2 at their flagellar pole, reinforcing the CtrA pathway, increasing CtrA ~P, promoting motility and preventing adhesive processes. In contrast, at the mother cell old pole after PdhS2 delocalization, DivJ kinase activity would dominate, the CtrA pathway would be inhibited and lower CtrA ~P levels would promote DNA replication, maintaining a sessile, non-motile state. Computational models of asymmetric cell development in *C. crescentus* support this notion, with the important caveat that phospho-DivK may not be distributed in a gradient, but is rather locally restricted [24, 25, 75].

### PdhS2 may regulate CtrA activity via an alternative route

**Fig. 7.**
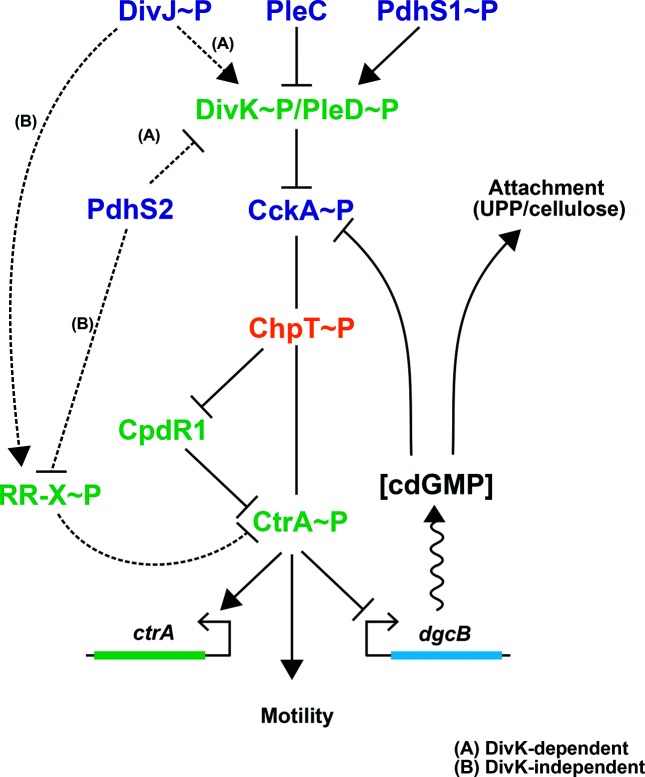
An alternative model for PdhS2 regulation of CtrA activity. Our data are consistent with PdhS2 intersecting the DivK–CtrA regulatory pathway at one of two points. Pathway A: canonical genetic model with PdhS2 interacting with DivK. The phosphorylation status of DivK then modulates CtrA activity through the CckA–ChpT–CtrA axis. Pathway B: DivK-independent model of CtrA regulation by PdhS2 through an unidentified response regulator, RR-X. Both routes to the regulation of CtrA activity ultimately affect the phosphorylation status of CtrA, affecting occupancy at CtrA-regulated promoters, and finally leading to inverse regulation of attachment (primarily through cdGMP pools) and separately motility. Regulatory proteins: blue text; histidine kinases; orange text, histidine phosphotransferase (Hpt), green text, response regulators. RR-X indicates a putative response regulator, yet to be identified.

The recognized architecture of the DivK–CtrA regulatory pathway in several alphaproteobacteria, coupled with our data demonstrating a genetic interaction between *divK* and *pdhS2* in *A. tumefaciens *, are consistent with PdhS2, primarily through its phosphatase activity, decreasing DivK phosphorylation, similar to what is predicted for the other PdhS-type kinase PleC (Fig. 7, model A). The *pdhS2* mutant phenotype is, however, in stark contrast to the other non-essential *A. tumefaciens* PdhS-type mutants and the *divK* mutant, which all cause cell branching [36]. How does PdhS2 regulate the same pathway so differently from the PdhS-type proteins? Possibly, spatial restriction of PdhS2 activity to the new poles of mother cells, which rapidly transition to become the old poles of newly formed daughter cells, imparts PdhS2 control of motility and attachment processes, without strongly influencing the budding process per se. Alternatively, PdhS2 may act via a different mechanism to influence CtrA activity.

An interesting possibility is that PdhS2 and DivK may work in parallel rather than in series to impact on CtrA activity and its target genes (Fig. 7, model B). The apparent epistasis of the *divK* mutation over the *pdhS2* mutation could result from the unfettered kinase activity of CckA in the absence of *divK*, which titrates the impact of the *pdhS2* mutation. Our results, in which the expression of wild-type and kinase-biased CckA (CckA^Y67D^) alleles in the Δ*pdhS2* mutant only modestly affect its mutant phenotypes, support this proposal (Fig. 3). The CckA^Y67D^ mutant was isolated as a spontaneous suppressor of the swimming deficiency of a *pleC* mutant [36]. Plasmid-borne ectopic expression of the CckA^Y67D^ effectively reversed *pleC* phenotypes, in contrast to the observation that it does not suppress *pdhS2* mutant phenotypes (Fig. 3). This suggests that PdhS2 does not act similarly to PleC to inhibit DivK phosphorylation. A plausible explanation is that PdhS2 control of CtrA activity is independent of DivK and CckA (Fig. 7, model B). Although uninhibited CckA kinase activity in the absence of *divK* can overcome the effect of the *pdhS2* mutation, perhaps the kinase-biased CckA^Y67D^ allele is insufficiently active to do so. In this model, PdhS2 intercepts the DivK–CtrA signalling axis at a node downstream or independent of CckA (Fig. 7). Our findings reveal that the phosphatase activity of PdhS2 is dominant in its impact on CtrA-dependent targets, suggesting that it dephosphorylates a response regulator that is itself inhibitory to CtrA activity. These models are currently being tested, but are more challenging due to the essentiality of many of the regulatory components in this domain of the pathway for *A. tumefaciens *, including DivJ itself.

## Supplementary Data

Supplementary File 1
