# Supplementary material for: Reciprocal control of motility and biofilm formation by the PdhS2 two-component sensor kinase of Agrobacterium tumefaciens
Source: Microbiology (Reading). 2019 Jan 8;165(2):146–62. doi: 10.1099/mic.0.000758 (PMC7003649; doi:10.1099/mic.0.000758)

SUPPLEMENTARY MATERIAL

Reciprocal control of motility and biofilm formation by the PdhS2 two-component sensor kinase of *Agrobacterium tumefaciens*

Jason E. Heindl, Daniel Crosby, Sukhdev Brar, John F. Pinto, Tiyan Singletary, Daniel Merenich, Justin L. Eagan, Aaron M. Buechlein, Eric L. Bruger, Christopher M. Waters and Clay Fuqua

Running title: *Agrobacterium PdhS2 regulates motility and biofilms*

1) Supplementary Figures – S1-S8

2) Supplementary Figure Legends

3) Supplementary Tables – S1-S3

4) Supplementary References

Figure S1. A combined kinase- and phosphatase-null PdhS2 mutant allele has little effect on biofilm formation or swimming motility. The ability of plasmid-borne expression of a kinase- and phosphatase- null allele of *pdhS2* (p-*pdhS2* (K^-P^-)) to complement the Δ*pdhS2* phenotypes was compared against the wild-type *pdhS2* allele (p-*pdhS2*). Biofilm formation (black bars) and swimming motility (white bars) were evaluated as in Figure 2. (^{a}) = $P < 0.05$ compared to the wild-type background with vector only; (^{b}) = $P < 0.05$ compared to the Δ*pdhS2* background with vector only. Statistical significance was determined using Student's *t* test.

Figure S2. Morphology of WT, Δ*divK*, Δ*pdhS2*, and Δ*divK* Δ*pdhS2* strains. Strains were grown to exponential phase in ATGN. Aliquots of cells were placed on top of an ATGN/1% agarose pad and imaged using phase contrast microscopy. (A), WT; (B) Δ*divK*; (C) Δ*pdhS2*; (D) Δ*divK* Δ*pdhS2*. Representative images are shown. Scale bar = 10 μm.

Figure S3. PleD regulation of swimming motility is epistatic to PleC. Swimming motility of the wild-type (WT) and indicated mutant strains was evaluated as described in Figure 2. (*) $P < 0.05$ compared to all strains.

Figure S4. Predicted CtrA-dependent promoters bearing one or more CtrA binding motifs. Promoter regions from select genes whose expression is upregulated (red) or downregulated (black) in the Δ*pdhS2* mutant background relative to the wild-type background. Predicted CtrA binding sites, as defined in the main text, are

indicated. Only those genes with a predicted full CtrA binding site are shown. Expression levels were determined either via microarray expression profiling or beta-galactosidase translational reporters (Tables 1 and 2 in the main text).

Figure S5. PdhS2 regulation of swimming motility is independent of diguanylate cyclase activity. Swimming motility of the wild-type (WT) and indicated mutant strains was evaluated as described in Figure 2. $P < 0.05$ compared to WT ([a]), ∆*pdhS2* ([b]), or corresponding diguanylate cyclase mutant ([c]).

Figure S6. PdhS2 does not affect global levels of cyclic-di-GMP. Cyclic-di-GMP levels were measured in whole cell extracts from equivalent ODs of the indicated strains. Data are from three independent experiments (N = 3).

Figure S7. A catalytically inactive DgcB modestly affects swimming motility. The effect on swimming motility of plasmid-borne expression of wild-type *dgcB* (p-*dgcB*) or a catalytic mutant allele of *dgcB* (p-*dgcB**) was evaluated. Expression of each *dgcB* allele was driven by the P_{lac} promoter. Swimming motility was evaluated as described in Figure 2. (*) = $P < 0.05$ compared to vector alone.

Figure S8. PdhS2 and DivJ are polarly localized in *A. tumefaciens*. Time-lapse microscopy of a C-terminal green fluorescent protein fusion to PdhS2 (A) and DivJ (B). Overlaid phase and fluorescent images, acquired sequentially on Nikon E800

fluorescence microscope with a CCD camera using the 100 X objective. Time between panels is 40 minutes. To the right of each image is a cartoon interpretation of the image.

Table S1. Strains used in this study

Species	Strain	Relevant Characteristics	Source
A. tumefaciens	C58	Nopaline type strain, pAtC58, pTiC58	[1]
A. tumefaciens	C58-JE001	Δ*dgcB* Δ*pdhS2* Δ*pleD* (ΔAtu1691 ΔAtu1888 ΔAtu1297)	This study
A. tumefaciens	C58-JE002	Δ*dgcB* Δ*pleD* (ΔAtu1691 ΔAtu1297)	This study
A. tumefaciens	C58-JEH076	Δ*pdhS2* (ΔAtu1888)	[2]
A. tumefaciens	C58-JEH128	Δ*pdhS2* Δ*pleD* (ΔAtu1888 ΔAtu1297)	This study
A. tumefaciens	C58-JEH130	Δ*pdhS2* Δ*dgcA* (ΔAtu1888 ΔAtu1257)	This study
A. tumefaciens	C58-JEH131	Δ*pdhS2* Δ*dgcB* (ΔAtu1888 ΔAtu1691)	This study
A. tumefaciens	C58-JEH132	Δ*pdhS2* Δ*dgcC* (ΔAtu1888 ΔAtu2179)	This study
A. tumefaciens	C58-JEH145	Δ*pdhS2* Δ*pleC* (ΔAtu1888 ΔAtu0982)	This study
A. tumefaciens	C58-JEH146	Δ*pdhS2* Δ*crdS* Δ*chvAB* Δ*cel* Δ*upp* Δ*exoA* (ΔAtu1888 ΔAtu3055-3057 ΔAtu2728-2730 ΔAtu3302-8187 ΔAtu1235-1240 ΔAtu4053)	This study

A. tumefaciens	C58-JEH147	Δ*pdhS2* Δ*upp* (ΔAtu1888 ΔAtu1235-1240)	This study
A. tumefaciens	C58-JEH148	Δ*pdhS2* Δ*cel* (ΔAtu1888 ΔAtu3302-8187)	This study
A. tumefaciens	C58-JEH149	Δ*pdhS2* Δ*crdS* (ΔAtu1888 ΔAtu3055-3057)	This study
A. tumefaciens	C58-JEH150	Δ*pdhS2* Δ*chvAB* (ΔAtu1888 ΔAtu2728-Atu2730)	This study
A. tumefaciens	C58-JEH151	Δ*pdhS2* Δ*exoA* (ΔAtu1888 ΔAtu4053)	This study
A. tumefaciens	C58-JEH153	Δ*divK* Δ*pdhS2* (ΔAtu1296 ΔAtu1888)	This study
A. tumefaciens	C58-JW2	Δ*pleC* (ΔAtu0982)	[2]
A. tumefaciens	C58-JW7	Δ*divK* (ΔAtu1296)	[2]
A. tumefaciens	C58-JW8	Δ*pleD* (ΔAtu1297)	[2]
A. tumefaciens	C58-JX100	Δ*crdS* (ΔAtu3055-3057)	[3]
A. tumefaciens	C58-JX101	Δ*chvAB* (ΔAtu2728-2730)	[3]
A. tumefaciens	C58-JX102	Δ*cel* (ΔAtu3302-8187)	[3]
A. tumefaciens	C58-JX111	Δ*crdS* Δ*chvAB* Δ*cel* Δ*upp* Δ*exoA* (ΔAtu3055-3057 ΔAtu2728-2730	[3]

		ΔAtu3302-8187 ΔAtu1235-1240 ΔAtu4053; "EPS^{-}")	
A. tumefaciens	C58-JX125	Δ*dgcA* (ΔAtu1257)	[4]
A. tumefaciens	C58-JX187	Δ*dgcB* (ΔAtu1691)	[4]
A. tumefaciens	C58-MLL2 A	Δ*exoA* (ΔAtu4053)	[5]
A. tumefaciens	C58-PMM26	Δ*upp* (ΔAtu1235-1240)	[3]
A. tumefaciens	C58-YW010	Δ*dgcC* (ΔAtu2179)	[4]
E. coli	S17-1 λ*pir*	RK2 *tra* regulon, *pir*, host for *pir*-dependent plasmids	[6]
E. coli	TOP10 F'	F'*lac*I^{q} Tn*10* (Tet^{R}) *mcr*A Δ(*mrr*-*hsd*RMS-*mcr*BC) Φ80*lacZ*ΔM15 Δ*lac*X74 *rec*A1 *ara*D139 Δ(*ara*-*leu*)7697 *gal*U *rps*L *end*A1 *nup*G	Thermo Fisher Scientific

Table S2. Plasmids used in this study

Plasmid name	Relevant characteristics	Source
pGEM-T Easy	PCR cloning vector; Amp^R	Promega
p*lacZ*/290	Broad host range plasmid carrying promoterless *lacZ* for transcriptional fusions; Tet^R	[7]
pNPTS138	ColE1 origin; *sacB*; Km^R	gift of M. Alley
pRA301	Broad host range plasmid carrying promoterless *lacZ* for translational fusions; $Spec^R$	[8]
pSRKGm	Broad host range vector containing P_{lac}; $lacI^q$; $lacZ\alpha^+$; Gm^R	[9]
p*ctrA*290	p*lacZ*/290 derivative with *C. crescentus ctrA* promoter	[10]
pDC001	pGEM-T Easy with full-length $pdhS2^{(CA811\text{-}812GC, A823G)}$ ($PdhS2^{His271A,Thr275Ala}$ mutant)	This study

pDC002	pSRKGm with full-length *pdhS2*$^{(CA811-812GC, A823G)}$ (PdhS2^{His271A,Thr275Ala} mutant)	This study
pGZ22	p*lacZ*/290 derivative with *C. crescentus ccrM* promoter	[11]
pJEH010	pSRKGm with full-length wild-type *cckA*	[2]
pJEH021	pGEM-T Easy with full-length *pdhS2*	[2]
pJEH026	pSRKGm with full-length *pdhS2*	[2]
pJEH030	pSRKGm with full-length Y674D *cckA* allele	[2]
pJEH040	pNPTS138 derivative with *pdhS2* SOE deletion fragment	[2]
pJEH052	pGEM-T Easy with *pdhS2* lacking a stop codon	This study
pJEH053	pGEM-T Easy with *gfpmut3*	This study

pJEH054	pGEM-T Easy with *divJ* lacking a stop codon	This study
pJEH060	pSRKGm with a *pdhS2*::*gfpmut3* translational fusion	This study
pJEH078	pSRKGm with a *divJ*::*gfpmut3* translational fusion	This study
pJEH091	pGEM-T Easy with full-length *pdhS2*$^{(CA811\text{-}812GC)}$ (PdhS2^{His271Ala} mutant)	This study
pJEH092	pSRKGm with full-length *pdhS2*$^{(CA811\text{-}812GC)}$ (PdhS2^{His271Ala} mutant)	This study
pJEH099	pGEM-T Easy with full-length *pdhS2*$^{(A823G)}$ (PdhS2^{Thr275Ala} mutant)	This study
pJEH102	pSRKGm with full-length *pdhS2*$^{(A823G)}$ (PdhS2^{Thr275Ala} mutant)	This study
pJEH113	pGEM-T Easy with *A. tumefaciens ccrM* promoter	This study

pJEH115	pGEM-T Easy with *A. tumefaciens ctrA* promoter	This study
pJEH119	pGEM-T Easy with *A. tumefaciens pdhS1* promoter	This study
pJEH121	pRA301 with *A. tumefaciens ccrM* promoter	This study
pJEH122	pRA301 with *A. tumefaciens ctrA* promoter	This study
pJEH124	pRA301 with *A. tumefaciens pdhS1* promoter	This study
pJEH141	pRA301 with 5’-TTAA-3’ → 5’-AATT-3’ mutation of CtrA binding motif in *A. tumefaciens* dgcB promoter	This study
pJFP006	pRA301 with 5’-TTAA-3’ → 5’-AATT-3’ mutation of CtrA binding motif in *A. tumefaciens* Atu3318 promoter	This study

pJW109	pNPTS138 derivative with *pleD* SOE deletion fragment	[2]
pJS70	p*lacZ*/290 derivative with *C. crescentus pilA* promoter	[12]
pJX158	pRA301 with *A. tumefaciens* Atu3318 promoter	[4]
pJX162	pRA301 with *A. tumefaciens dgcB* promoter	[4]
pJX520	pSRKGm with full-length *dgcB*	[4]
pJX521	pSRKGm with full-length *dgcB*$^{A767C, A770C}$ (DgcB$^{EE256-257AA}$ mutant)	[4]
pJX802	pNPTS138 derivative with *dgcB* SOE deletion fragment	[4]
pJZ383	pPZP201 derivative with P_{tac}::*gfpmut3*; Spec^R	[13]

Table S3. Primers used in this study

Primer	Sequence (5' – 3')	Use
JEH65	GAAGAACATATGAGTAAAAGCGTCAGCA	cloning *pdhS2* with NdeI site
JEH85	GATTTCGCGCGATCCCTTCGA	Internal primer for *pdhS2* locus
JEH87	GAGCAGATGCTGGCCGGA	Internal primer for *pdhS2* locus
JEH100	GCTCTGTTGAAGGCGGCCAA	External primer for *pdhS2* locus
JEH113	GCCGGTTTCATGCACACGCA	External primer for *pdhS2* locus
JEH146	GAAGAAGCTAGCGGCGAAAGACCGCCGG	cloning *pdhS2* w/o STOP and with NheI site
JEH147	GAAGAACATATGAGAGAAAAAGCGGTCGCA	cloning *divJ* with NdeI site
JEH148	GAAGAAGCTAGCGGCGATTTTCGCTTTCGCGG	cloning *divJ* w/o STOP and with NheI site
JEH149	GAAGAAGGTACCTTATTTGTATAGTTCATCCATGCCA	cloning *gfpmut3* with KpnI site
JEH150	GAAGAAGCTAGCATGAGTAAAGGAGAAGAACTT	cloning *gfpmut3* with NheI site
JEH245	CGTGCGCAGCTCGgcCGACATGGAAGCG	*pdhS2*$^{CA811\text{-}812GC}$ mutagenesis
JEH246	CGCTTCCATGTCGgcCGAGCTGCGCACG	*pdhS2*$^{CA811\text{-}812GC}$ mutagenesis
JEH261	CGCACGAGCTGCGCgCGCCGCTCAACGC	*pdhS2*^{A823G} mutagenesis
JEH262	GCGTTGAGCGGCGcGCGCAGCTCGTGCG	*pdhS2*^{A823G} mutagenesis
JEH282	GGTACCTGCCAGAATCGTTGCT	cloning *ccrM* promoter region, +222 bp to -9 bp from translational start, with KpnI site
JEH284	AAGCTTTGCTGCCATTGGTACT	cloning *ccrM* promoter region, +222 bp to -9 bp from translational start, with HindIII site
JEH285	GGTACCTTAACCTTTCGTTTACGGGCA	cloning *ctrA* promoter region, +328 bp to -9 bp from translational start, with KpnI site
JEH287	CTGCAGAACCCGCATAATTATCCCCTTT	cloning *ctrA* promoter region, +328 bp to -9 bp from

		translational start, with PstI site
JEH291	GGTACCATTTGCAAGTGCCTCTT	cloning *pdhS1* promoter region, +264 bp to -9 bp from translational start, with KpnI site
JEH293	AAGCTTGGCGGGCATGTCGAAA	cloning *pdhS1* promoter region, +264 bp to -9 bp from translational start, with HindIII site

REFERENCES

1. **Watson B, Currier TC, Gordon MP, Chilton MD, Nester EW**. Plasmid required for virulence of *Agrobacterium tumefaciens*. *Journal of Bacteriology* 1975;123(1):255-264.

2. **Kim J, Heindl JE, Fuqua C**. Coordination of division and development influences complex multicellular behavior in *Agrobacterium tumefaciens*. *PloS one* 2013;8(2):e56682. doi: 10.1371/journal.pone.0056682

3. **Xu J, Kim J, Danhorn T, Merritt PM, Fuqua C**. Phosphorus limitation increases attachment in *Agrobacterium tumefaciens* and reveals a conditional functional redundancy in adhesin biosynthesis. *Res Microbiol* 2012;163(9-10):674-684. doi: 10.1016/j.resmic.2012.10.013

4. **Xu J, Kim J, Koestler BJ, Choi JH, Waters CM et al.** Genetic analysis of *Agrobacterium tumefaciens* unipolar polysaccharide production reveals complex integrated control of the motile-to-sessile switch. *Molecular microbiology* 2013;89(5):929-948. doi: 10.1111/mmi.12321

5. **Tomlinson AD, Ramey-Hartung B, Day TW, Merritt PM, Fuqua C**. *Agrobacterium tumefaciens* ExoR represses succinoglycan biosynthesis and is required for biofilm formation and motility. *Microbiology* 2010;156(Pt 9):2670-2681. doi: 10.1099/mic.0.039032-0

6. **de Lorenzo V, Timmis KN**. Analysis and construction of stable phenotypes in Gram-negative bacteria with Tn5- and Tn10-derived minitransposons. *Methods Enzymol* 1994;235:386-405. doi: 10.1016/0076-6879(94)35157-0

7. **Gober JW, Shapiro L**. A developmentally regulated *Caulobacter* flagellar promoter is activated by 3' enhancer and IHF binding elements. *Mol Biol Cell* 1992;3(8):913-926. doi: 10.1091/mbc.3.8.913

8. **Akakura R, Winans SC**. Constitutive mutations of the OccR regulatory protein affect DNA bending in response to metabolites released from plant tumors. *The Journal of biological chemistry* 2002;277(8):5866-5874. doi: 10.1074/jbc.M110555200

9. **Khan SR, Gaines J, Roop RM, 2nd, Farrand SK**. Broad-host-range expression vectors with tightly regulated promoters and their use to examine the influence of TraR and TraM expression on Ti plasmid quorum sensing. *Applied and environmental microbiology* 2008;74(16):5053-5062. doi: 10.1128/AEM.01098-08

10. **Domian IJ, Reisenauer A, Shapiro L**. Feedback control of a master bacterial cell-cycle regulator. *Proceedings of the National Academy of Sciences of the United States of America* 1999;96(12):6648-6653. doi: 10.1073/pnas.96.12.6648

11. **Stephens CM, Zweiger G, Shapiro L**. Coordinate cell cycle control of a *Caulobacter* DNA methyltransferase and the flagellar genetic hierarchy. *Journal of bacteriology* 1995;177(7):1662-1669. doi: 10.1128/jb.177.7.1662-1669.1995

12. **Skerker JM, Shapiro L**. Identification and cell cycle control of a novel pilus system in *Caulobacter crescentus*. *The EMBO journal* 2000;19(13):3223-3234. doi: 10.1093/emboj/19.13.3223

13. **Ramey BE, Matthysse AG, Fuqua C**. The FNR-type transcriptional regulator SinR controls maturation of *Agrobacterium tumefaciens* biofilms. *Molecular microbiology* 2004;52(5):1495-1511. doi: 10.1111/j.1365-2958.2004.04079.x

Figure S1

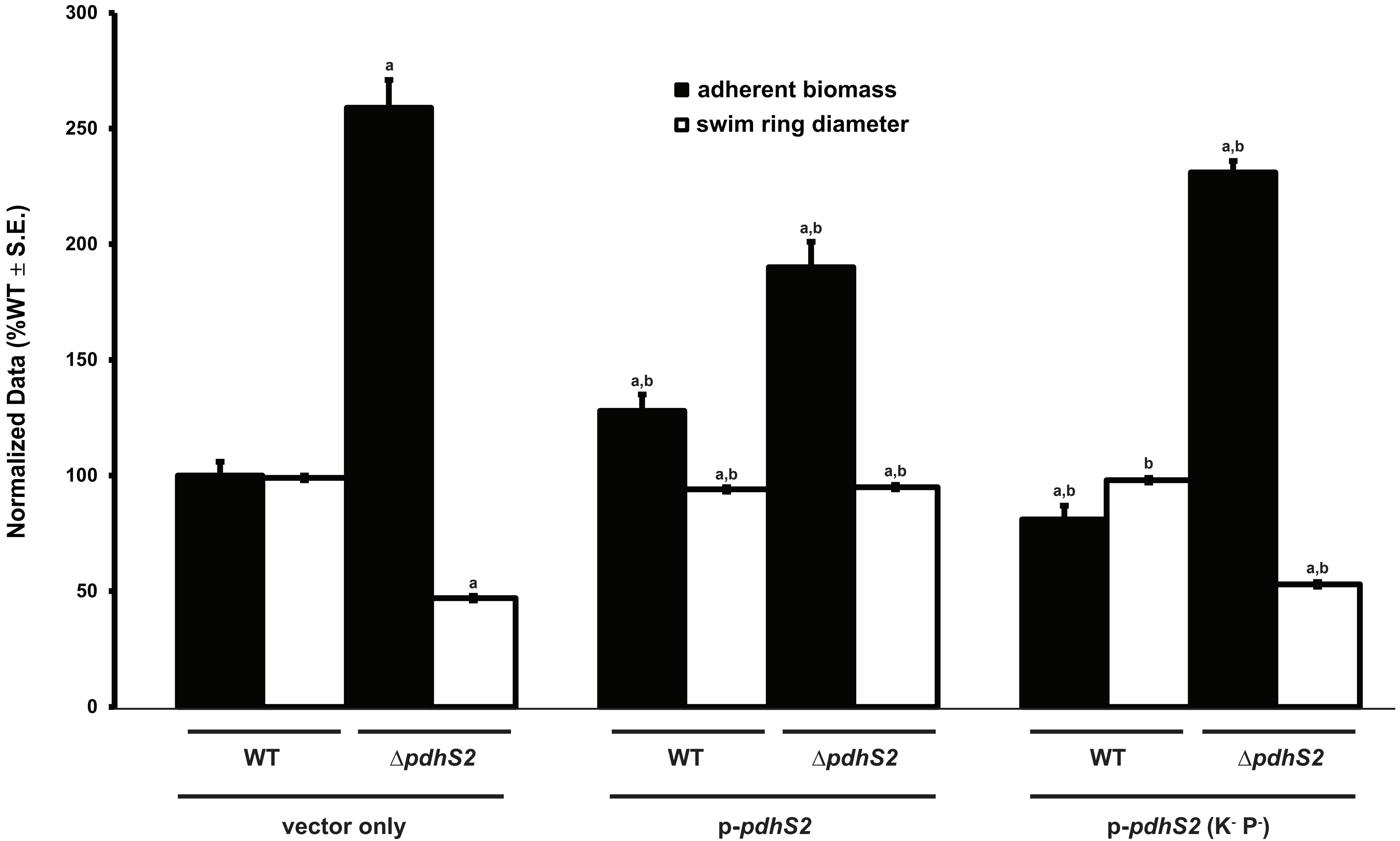

Normalized Data (%WT ± S.E.)
300
250
200
150
100
50
0
a
a
a,b
a,b
a,b
a,b
a,b
b
a,b
a,b
adherent biomass
swim ring diameter
WT
ΔpdhS2
WT
ΔpdhS2
WT
ΔpdhS2
vector only
p-pdhS2
p-pdhS2 (K- P-)

Figure S2

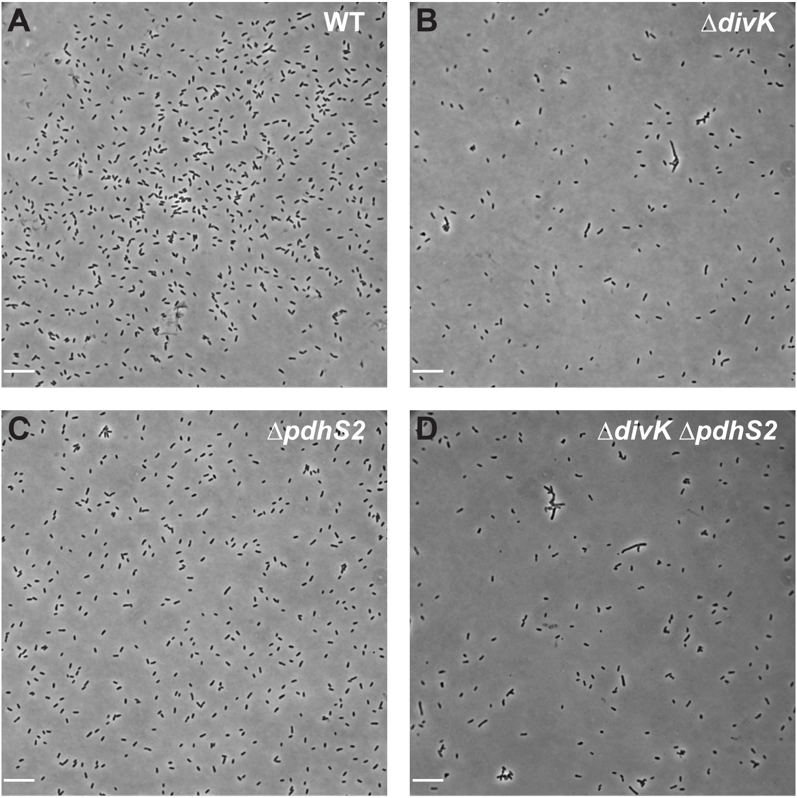

Figure S3

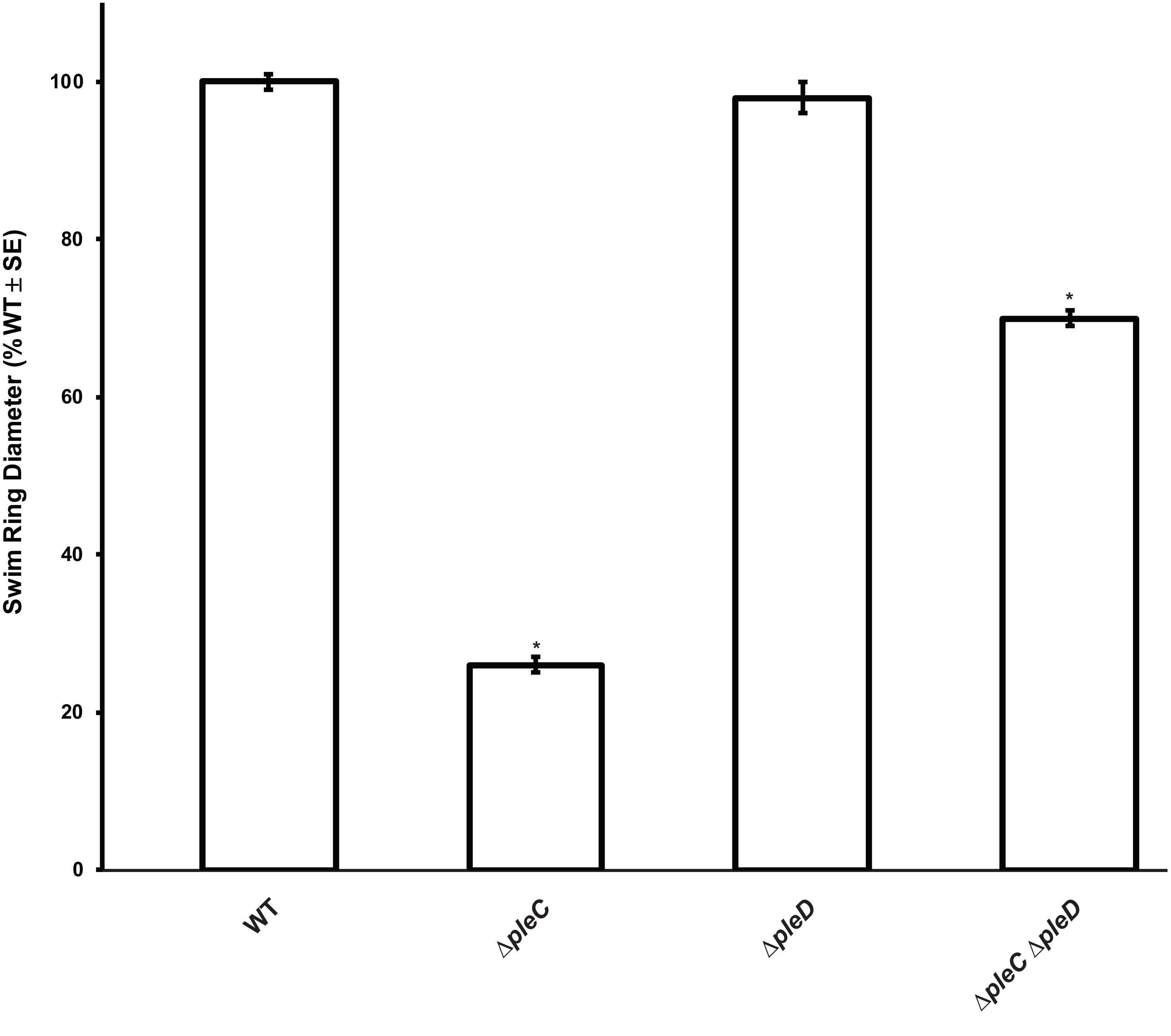

Swim Ring Diameter (% WT ± SE)
0
20
40
60
80
100
*
*
WT
ΔpleC
ΔpleD
ΔpleC ΔpleD

Figure S4

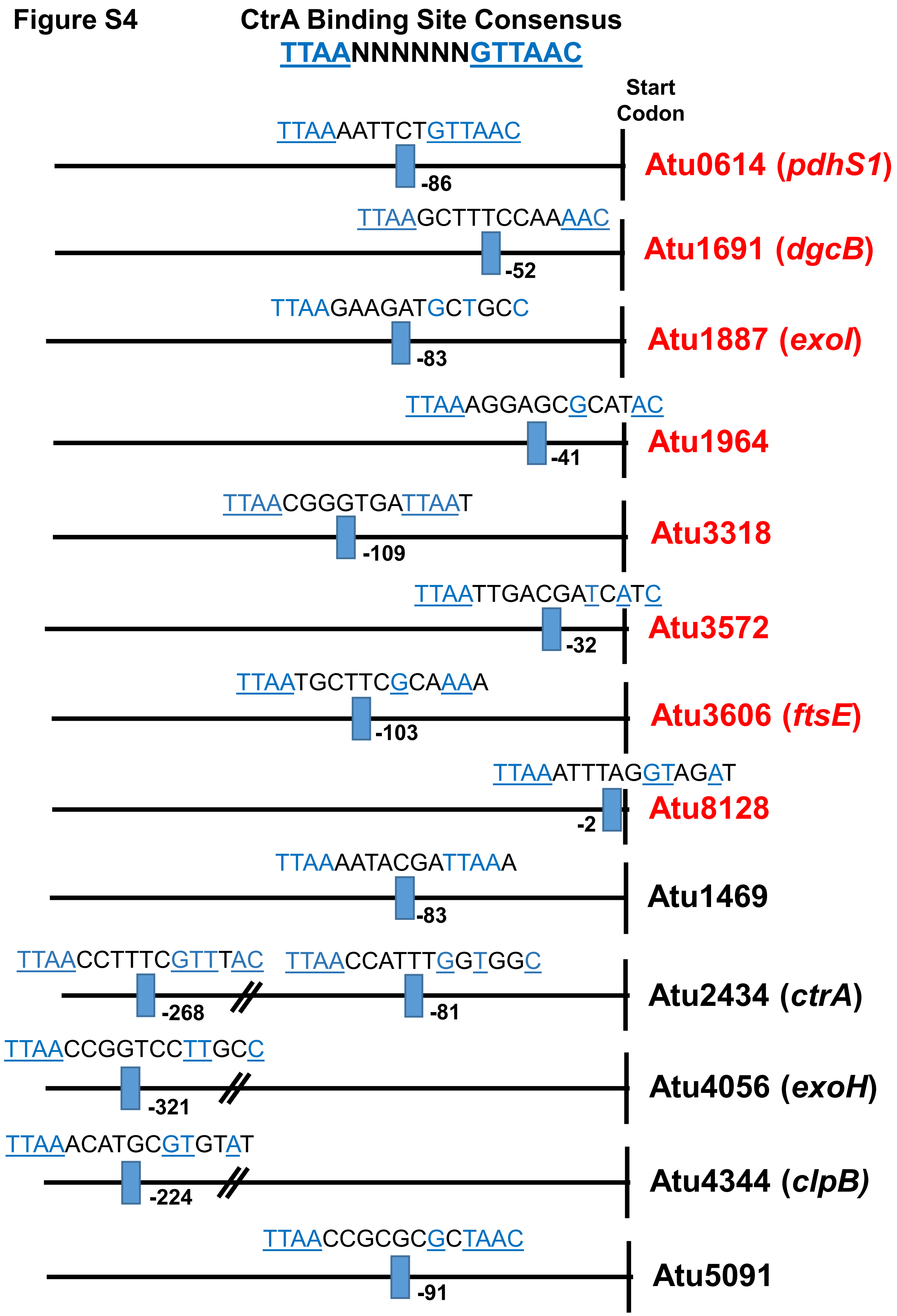
CtrA Binding Site Consensus
TTAANNNNNNGTTAAC
Start Codon
TTAAAATTCTGTTAAC
-86
Atu0614 (pdhS1)
TTAAGCTTTCCAAAAC
-52
Atu1691 (dgcB)
TTAAGAAGATGCTGCC
-83
Atu1887 (exoI)
TTAAAGGAGCGCATAC
-41
Atu1964
TTAACGGGTGATTAAT
-109
Atu3318
TTAATTGACGATCATC
-32
Atu3572
TTAATGCTTCGCAAAA
-103
Atu3606 (ftsE)
TTAAATTTAGGTAGAT
-2
Atu8128
TTAAAATACGATTAAA
-83
Atu1469
TTAACCTTTCGTTTAC
TTAACCATTTGGTGGC
-268
-81
Atu2434 (ctrA)
TTAACCGGTCCTTGCC
-321
Atu4056 (exoH)
TTAAACATGCGTGTAT
-224
Atu4344 (clpB)
TTAACCGCGCGCTAAC
-91
Atu5091

Figure S5

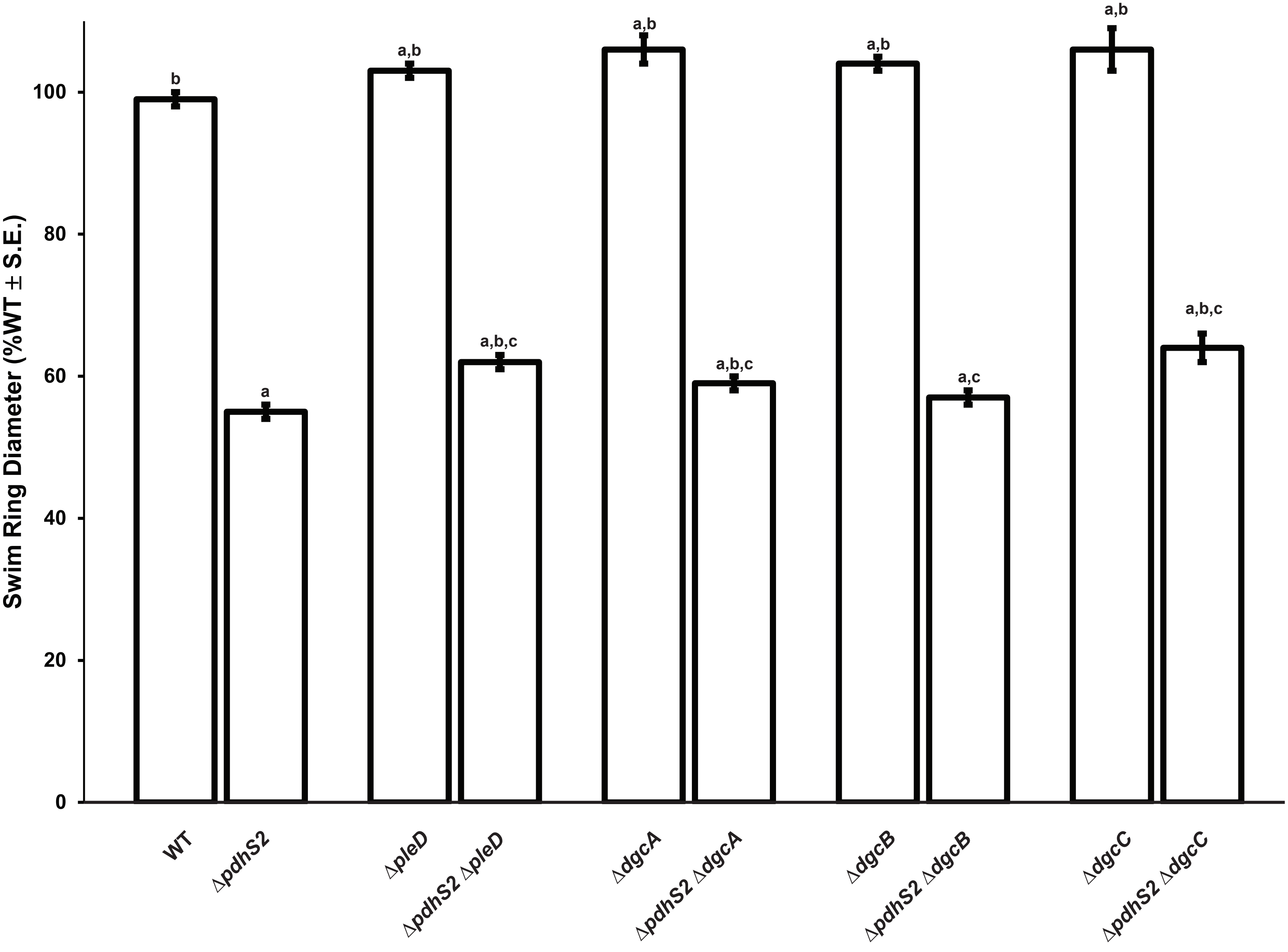

Swim Ring Diameter (%WT ± S.E.)
0
20
40
60
80
100
b
a
a,b
a,b,c
a,b
a,b,c
a,b
a,c
a,b
a,b,c
WT
ΔpdhS2
ΔpleD
ΔpdhS2 ΔpleD
ΔdgcA
ΔpdhS2 ΔdgcA
ΔdgcB
ΔpdhS2 ΔdgcB
ΔdgcC
ΔpdhS2 ΔdgcC

Figure S6

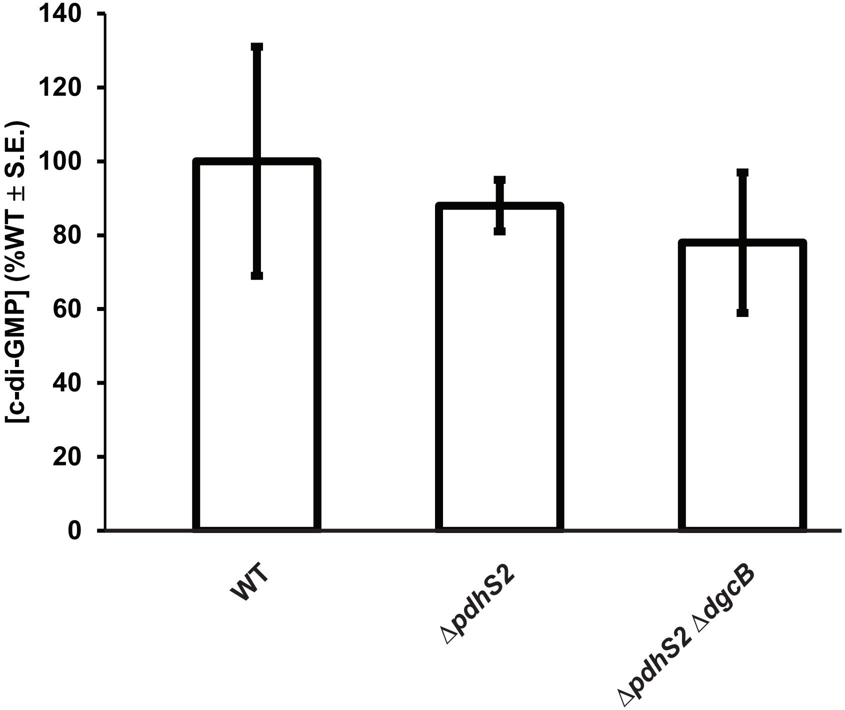

Figure S7

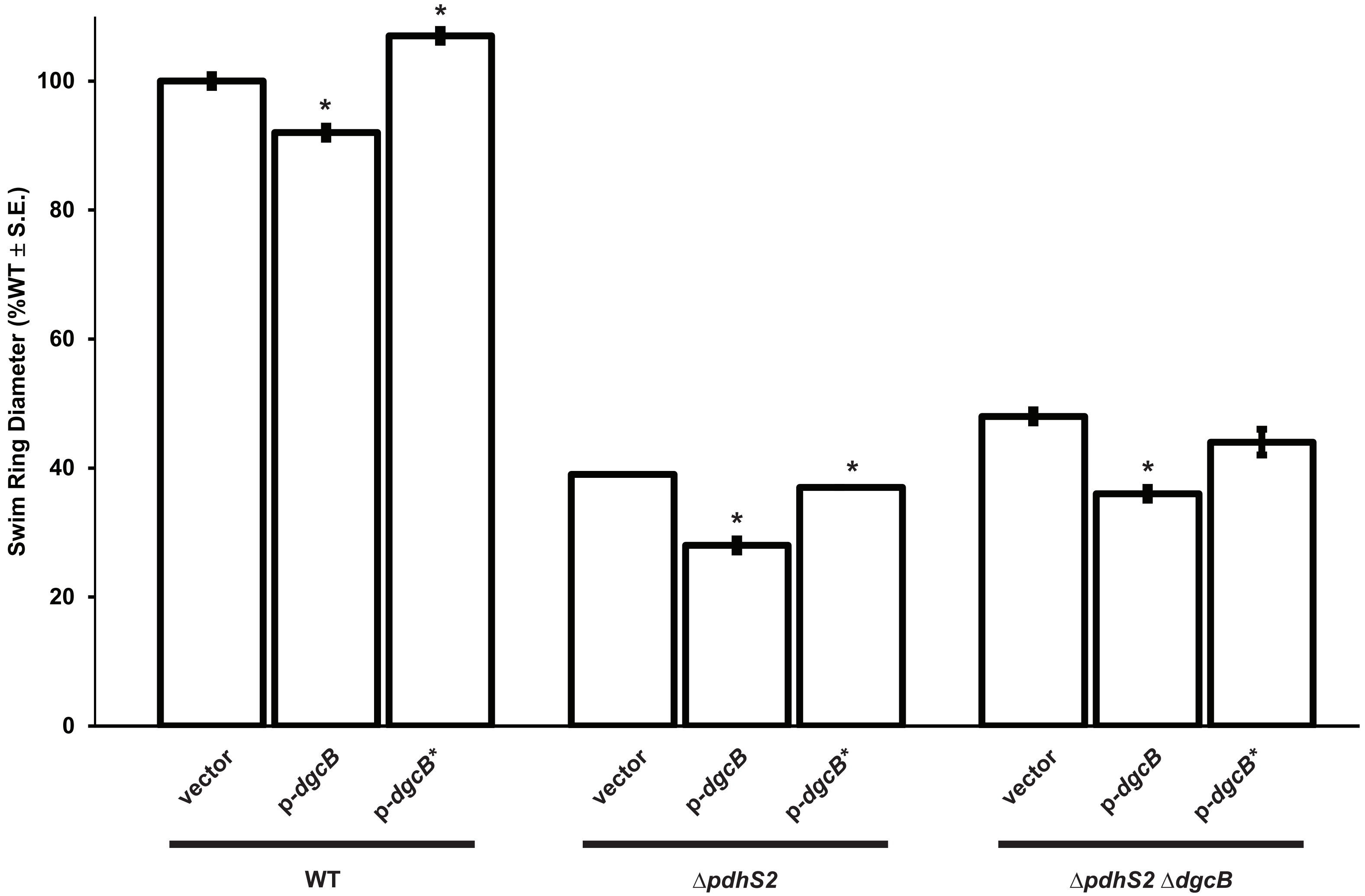

Swim Ring Diameter (%WT ± S.E.)
0
20
40
60
80
100
*
*
*
*
*
vector
p-dgcB
p-dgcB*
vector
p-dgcB
p-dgcB*
vector
p-dgcB
p-dgcB*
WT
ΔpdhS2
ΔpdhS2 ΔdgcB

Figure S8

A

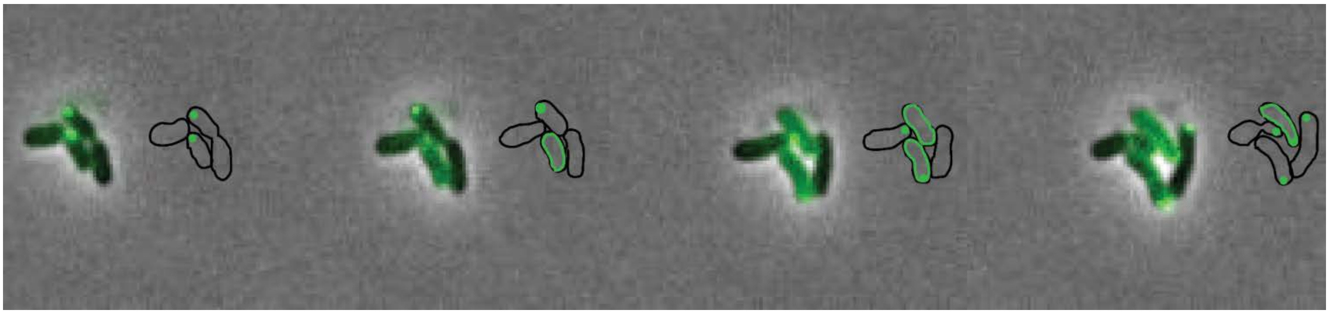

B

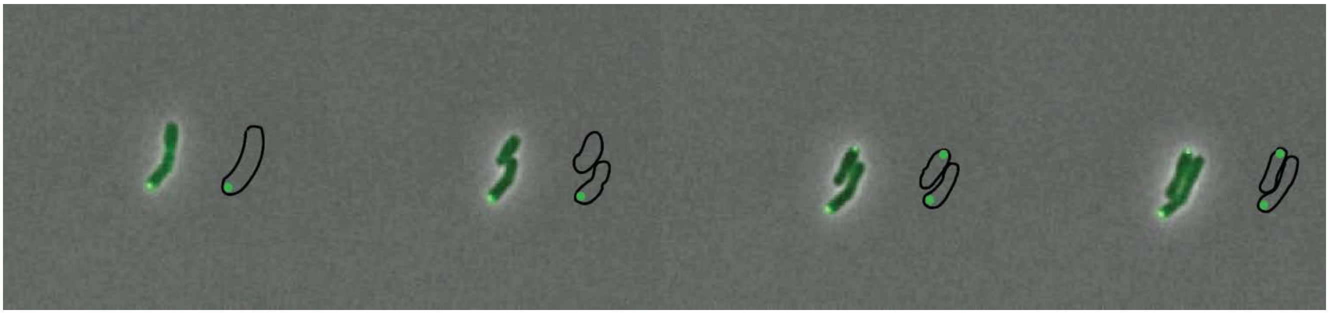

Supplement: Supplementary File 1 [file mic-165-146-s001.pdf]
